# Comparative effectiveness and pharmacological fingerprints of indobufen versus rivaroxaban in patients with chronic kidney disease: a single-center, real-world study

**DOI:** 10.3389/fphar.2025.1694163

**Published:** 2025-11-19

**Authors:** Lijun Zhang, Mingbo Liu, Tingting Huang, He Zhang, Chuanfu Huang, Zhenbin Pan, Zhao Chen, Jun Ning, Jiameng Tang

**Affiliations:** 1 Department of Nephrology, The First People’s Hospital of Qinzhou, The Tenth Affiliated Hospital of Guangxi Medical University, Qinzhou, Guangxi, China; 2 Department of Laboratory Medicine, The First People’s Hospital of Qinzhou, The Tenth Affiliated Hospital of Guangxi Medical University, Qinzhou, Guangxi, China; 3 Department of Medical Statistics, The First People’s Hospital of Qinzhou, The Tenth Affiliated Hospital of Guangxi Medical University, Qinzhou, Guangxi, China

**Keywords:** chronic kidney disease (CKD), indobufen, rivaroxaban, real-world study, large language model (LLM), linear mixed model (LMM)

## Abstract

**Introduction:**

Antithrombotic management in Chronic Kidney Disease (CKD) is a clinical dilemma. This study aimed to empirically evaluate the “*de facto* interchangeability” of the antiplatelet indobufen and the anticoagulant rivaroxaban by comparing their real-world effectiveness and safety in hospitalized CKD patients.

**Methods:**

In this retrospective cohort study (2020-2024), we analyzed CKD patients treated with indobufen or rivaroxaban. A multi-stage analysis first used machine learning to assess baseline cohort comparability, overcoming limitations of p-value-based tests. Subsequently, a Linear Mixed Model (LMM), adjusted for confounders including polypharmacy, assessed independent drug effects on in-hospital thrombosis, hemorrhage, and longitudinal laboratory markers.

**Results:**

Machine learning demonstrated the clinical comparability of the indobufen and rivaroxaban cohorts. The incidence of in-hospital thrombosis was numerically lower in the indobufen group (3.65% vs. 7.58%; *P* = 0.101), while hemorrhage rates were similar (2.19% vs. 2.27%; *P* = 1). The LMM analysis, beyond verifying indobfen’s expected antiplatelet activity (modulating MPV, PDW), revealed pleiotropic effects (increased prealbumin, HDL-C) and a significant reduction in urine occult blood (*P* < 0.001), suggesting renal safety. Notably, the model demonstrated that apparent effects on hemoglobin and eGFR were attributable to confounding by co-medications, not a direct drug effect.

**Conclusion:**

In this real-world CKD cohort, indobufen and rivaroxaban demonstrated comparable clinical effectiveness and safety. Combining machine learning with longitudinal models helps to statistically adjust for complex confounders like polypharmacy, thereby providing a more robust estimate of a drug’s independent effect.

## Introduction

Chronic Kidney Disease (CKD) has emerged as a formidable global public health threat, characterized by high morbidity and a rapidly escalating mortality rate. According to the Global Burden of Disease Study 2023, CKD was responsible for 1.52 million deaths worldwide in 2023, a staggering 90.9% increase since 2000 (GBD, 2023 Causes of Death Collaborators, 2025). Within this challenging context, antithrombotic management is particularly problematic, limited by a narrow therapeutic window and potential nephrotoxicity (Saeed et al., 2024; Cases et al., 2021; Addisu et al., 2025). Indeed, CKD represents a paradoxical state with a concurrently elevated risk of both thrombosis and bleeding (Wang et al., 2023; Montomoli et al., 2024). On one hand, CKD constitutes a prethrombotic state driven by key mechanisms, including: endothelial dysfunction from uremia and inflammation; elevated levels of procoagulant factors (e.g., fibrinogen); and platelet hyperreactivity with increased release of procoagulant microparticles (MPs) (Alhousani et al., 2021; Noumegni et al., 2022). This sterile inflammation, mediated by the activation of the NLRP3 inflammasome by damage-associated molecular patterns (DAMPs), is a key driver of podocyte injury and renal fibrosis, accelerating CKD progression (Datta et al., 2025b; Datta et al., 2025a). On the other hand, platelet dysfunction caused by uremic toxins, coupled with the reduced metabolic clearance of anticoagulant drugs in patients with renal insufficiency, also significantly increases the risk of bleeding complications (Tham et al., 2024; Li et al., 2025). This dual risk makes antithrombotic treatment decisions in patients with CKD exceptionally challenging. As traditional injectable anticoagulants are ill-suited for long-term outpatient management (Almajdi et al., 2023; Expert Consensus Working Group, 2023) there is a pressing need to optimize the risk-benefit balance of oral antithrombotic agents in this complex population (Cases et al., 2021; Lip et al., 2021; Lau et al., 2022).

This study aims to conduct a head-to-head comparison of indobufen and rivaroxaban. The rationale for this comparison originates not from traditional pharmacological theory but from our observation of clinical equipoise. In patients with CKD, traditional antithrombotic agents have significant limitations: aspirin has failed to definitively reduce ischemic risk while increasing bleeding events (Wolfe et al., 2025; Stefanini et al., 2021; Festa et al., 2023), and the clinical applicability of warfarin is restricted by its narrow therapeutic window, a higher bleeding risk compared to Direct Oral Anticoagulants (DOACs), and the need for frequent INR monitoring (Cohen et al., 2022; Lau et al., 2022). Consequently, clinical focus has shifted to alternatives like clopidogrel (Gorog et al., 2023) and the FXa inhibitor rivaroxaban, whose indications have expanded (Ten Cate et al., 2021; Gao and Rahman, 2022). Indobufen, a reversible cyclooxygenase-1 (COX-1) inhibitor, is widely used in East Asia for aspirin-intolerant patients. Unlike aspirin’s irreversible inhibition, indobufen’s effect is transient, with platelet function recovering within 24 h. This theoretically confers better gastrointestinal tolerability and a lower bleeding risk (Dai C. et al., 2024; Jiang et al., 2025).

While studies have compared indobufen with aspirin and clopidogrel (Liu et al., 2024; Cavalcante et al., 2025), meta-analyses indicate its efficacy in preventing ischemic events is comparable to aspirin’s in cardiovascular settings (e.g., post-PCI/CABG), but with a significantly lower bleeding risk (Dai C. et al., 2024; Luo et al., 2024; Ren et al., 2024). This safety advantage may stem from its reversible, non-competitive inhibition of COX-1, which helps preserve gastric mucosal function while maintaining its antiplatelet effect (Liu et al., 2023). This favorable risk-benefit profile makes indobufen a promising option for patients with nephrotic syndrome (NS), who exhibit high thrombotic risk and a high bleeding propensity, partially attributed to gastrointestinal mucosal edema. However, existing research has focused on cardiovascular and stroke populations, largely neglecting its use in CKD. This study therefore aims to compare the real-world efficacy and safety of these functionally equivalent drugs, providing immediate clinical evidence and laying the foundation for future prospective trials and mechanistic explorations.

## Materials and methods

### Study design and data source

This study employed a retrospective cohort design, utilizing real-world data sourced from our institution’s Hospital Information System (HIS) and Laboratory Information System (LIS). The dataset included all patients hospitalized for Chronic Kidney Disease (CKD) between January 2020 and December 2024. Given the retrospective nature of the study and its focus on patients with specific clinical diagnoses, a separate cohort of healthy subjects was not enrolled to serve as a negative control group. Instead, all comparative analyses were conducted between different subgroups within the defined patient populations.

Inclusion criteria were as follows: (1) patients hospitalized for CKD who received antithrombotic therapy during their stay; and (2) patients who met at least one of the following conditions: ① presence of a hypercoagulable state during hospitalization or at discharge; ② occurrence of a new thrombotic event after the initiation of antithrombotic therapy; ③ occurrence of a bleeding event during hospitalization; or ④ presence of a bleeding tendency at discharge.

Exclusion criteria included: (1) age <12 years, to avoid confounding from developmental differences in pathophysiology and drug metabolism specific to pediatric populations; (2) total length of hospital stay <72 h, to ensure a sufficient observation period for the assessment of treatment effects and clinical outcomes; (3) significant missing data in key baseline characteristics, laboratory results, or medication records, as this would preclude a reliable assessment and introduce significant bias; and (4) receipt of combination antithrombotic therapy (i.e., concurrent use of two or more anticoagulant or antiplatelet agents) during hospitalization, in order to isolate the effects of the specific monotherapy under investigation and avoid confounding. For subsequent longitudinal analyses (e.g., paired t-tests, LMM), a complete-case approach was used, including only subjects with data available at both pre- and post-treatment time points, as reflected by the varying sample sizes (*n*) in the results tables.

### Data extraction and validation framework

To ensure high data fidelity, we developed a custom hybrid framework for data extraction from electronic medical records (EMRs). This system combined a Large Language Model (LLM) for identifying complex clinical events (e.g., thrombosis, bleeding) with a dual-pipeline validation process for structured data. All extracted events were temporally sequenced to distinguish pre-treatment from post-treatment onset and were manually adjudicated by two clinical researchers. A comprehensive description of the framework is provided in Supplementary Text S1.

### Criteria for event adjudication and status assessment

The diagnostic criteria for thrombotic and bleeding events were based on established international guidelines (e.g., ISTH, CTCAE v5.0). We also defined criteria for assessing patient status, specifically “Hypercoagulable State” (Chinese expert consensus on the application of anticoagulant technology in renal replacement therapy for critical patients (2023), 2023) and “Bleeding Tendency” (Jiang et al., 2023). The detailed operational definitions for all events and the specific laboratory criteria for these states are provided in Supplementary Text S1.

### Extraction and processing of laboratory results

To standardize the analysis of longitudinal laboratory data, we extracted results from predefined temporal windows corresponding to admission, discharge, 1/3/6-month follow-ups, and periods immediately pre- and post-treatment. For each window, a single representative data point was selected based on a consistent rule. The detailed definitions of these time windows and the selection criteria are provided in Supplementary Text S1.

### Analysis of clinical patterns and determinants of oral antithrombotic drug selection

This study employed a multi-stage analytical strategy to first identify clinical patterns associated with a hypercoagulable state and then to elucidate the factors driving the selection of specific oral antithrombotic agents. First, we utilized association rule mining with the Apriori algorithm (implemented in the R package “arules”) to identify clinical features and medication combinations that co-occurred with a hypercoagulable state (Si et al., 2022). Next, the analysis focused on the selection of specific oral antithrombotic agents (rivaroxaban, indobufen, and clopidogrel). We initially screened for clinical features associated with the choice of these drugs by performing a differential analysis of baseline characteristics, with an *p*-value <0.05 considered significant. Subsequently, LASSO (Least Absolute Shrinkage and Selection Operator) regression was employed to perform feature reduction and refine the selection of key predictors (Luo et al., 2023).

### Assessment of efficacy, adverse events, and Ancillary Effects

(1) Assessment of Treatment Efficacy: The incidence of new-onset thrombotic events during the treatment period or within specified post-treatment observation periods, and the presence of a hypercoagulable state during the discharge phase; (2) Assessment of Adverse Events: The incidence of new-onset bleeding events and the presence of a bleeding tendency. Both outcomes were evaluated during the treatment period and within specified post-treatment observation periods; (3) Analysis of Ancillary Effects on Laboratory Parameters:First, paired t-tests were used to compare the pre- and post-treatment values for each laboratory indicator. Subsequently, a Linear Mixed Model (LMM), implemented using the R package “lme4”, was constructed to provide a more robust assessment of the drug’s impact over time (Jimba et al., 2024; Yuan et al., 2025).

### Statistical analyses

All analyses were conducted using R (v4.4.0) and Python (v3.13.0). Continuous variables were expressed as mean ± SD or median (IQR) and compared using ANOVA or Kruskal–Wallis tests as appropriate. Categorical variables were presented as n (%) and compared using Chi-squared or Fisher’s exact tests. P-values for multiple comparisons were adjusted using the Benjamini–Hochberg FDR method. A two-sided P-value <0.05 was considered statistically significant.

## Result

### Patient baseline characteristics and description

**FIGURE 1:**
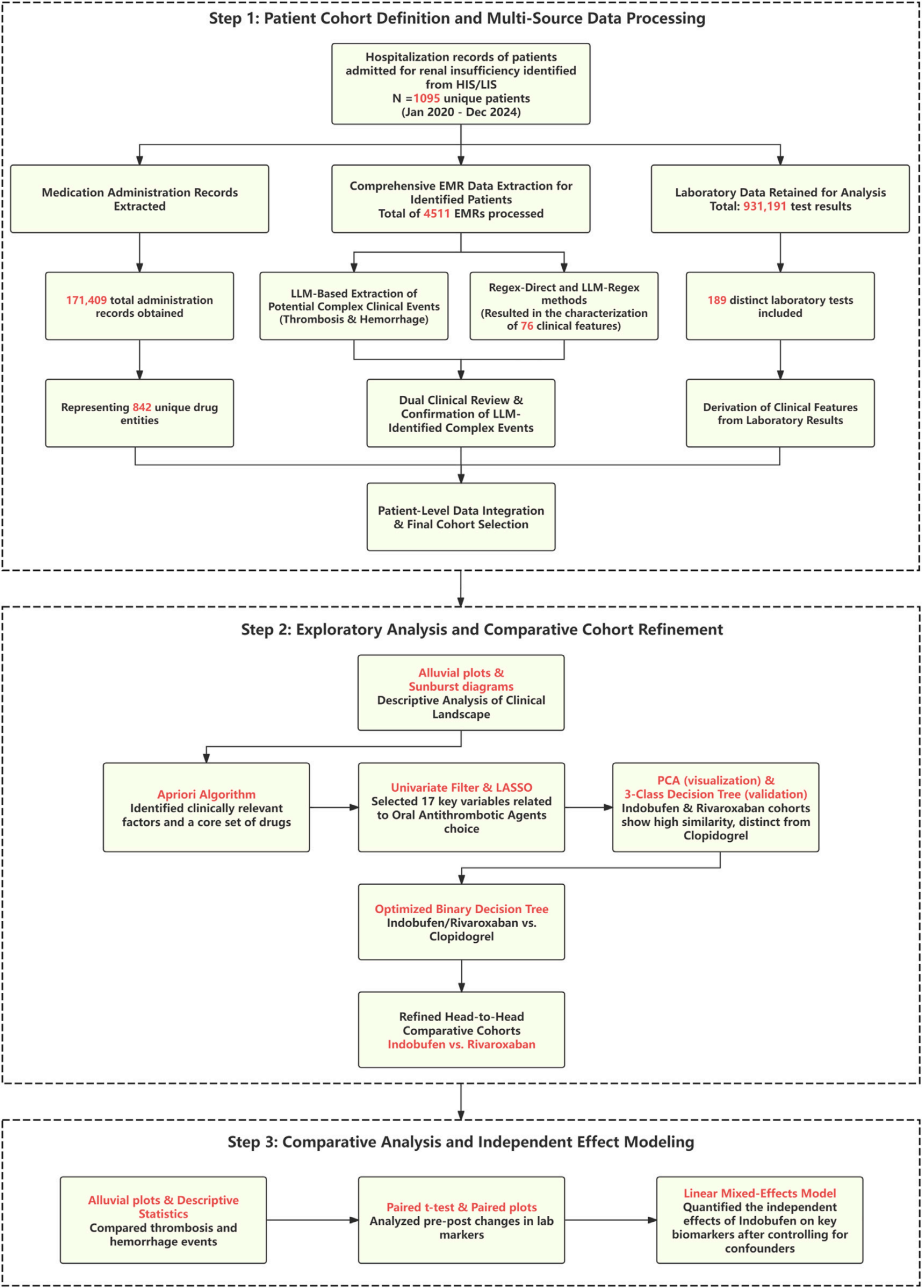
Flowchart of the study design and analytical workflow. The process begins with the definition of the patient cohort and the extraction of multi-source data using methods including Large Language Models (LLMs). Subsequently, a machine learning pipeline (including Apriori, LASSO, PCA, and decision trees) is used to refine the analysis to a head-to-head comparison of Indobufen and Rivaroxaban. Finally, a Linear Mixed Model (LMM) is applied to assess the independent effects of the target medication on clinical outcomes and laboratory parameters.

**FIGURE 2:**
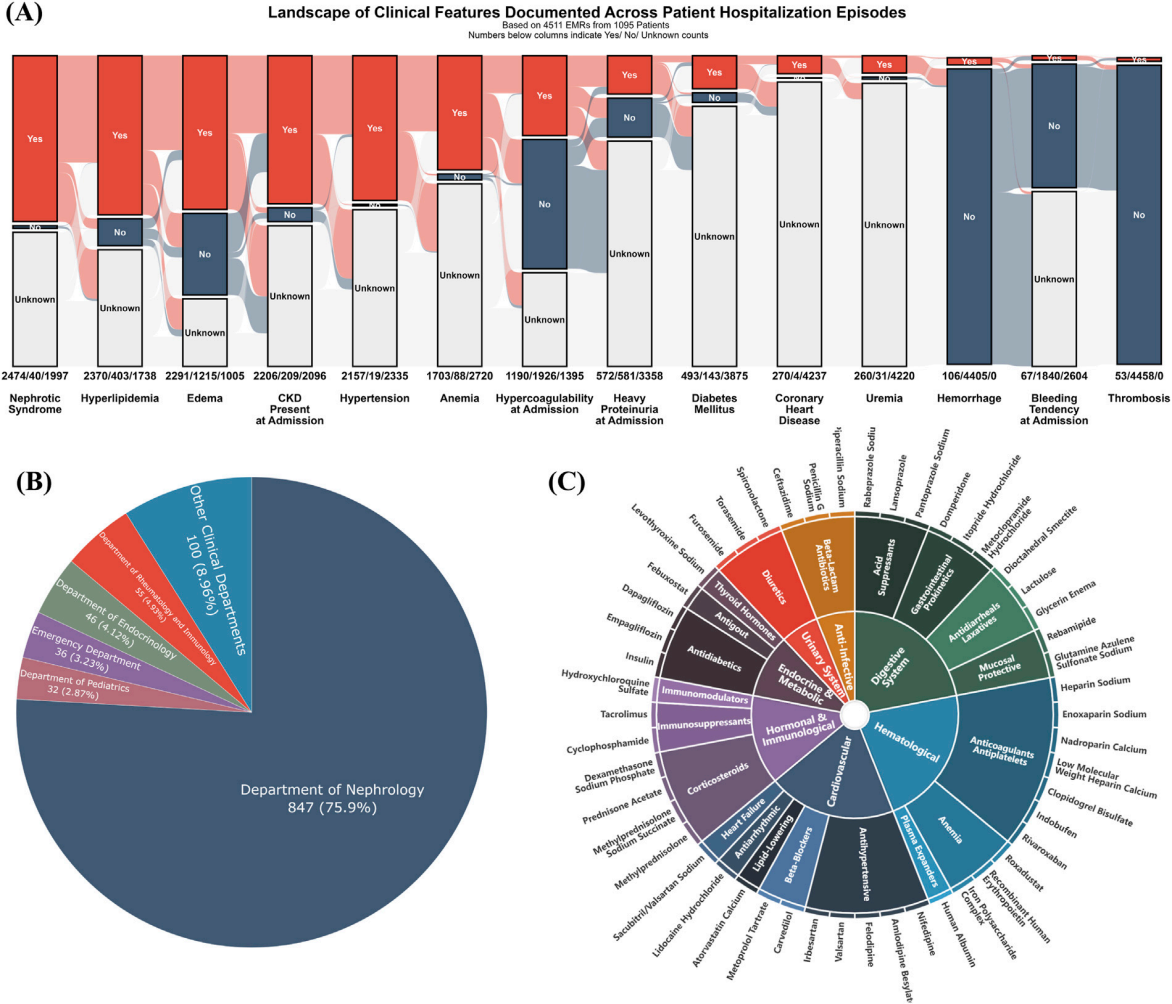
Baseline clinical landscape and medication profile of the study cohort. **(A)** Alluvial plot illustrating the prevalence of key clinical features, with counts for “Yes,” “No,” and “Unknown” statuses indicated across all 4,511 hospitalization events; **(B)** Pie chart depicting patient distribution by clinical department, predominantly Nephrology (75.9%); **(C)** Sunburst diagram illustrating the hierarchical classification of medications, focusing on the top 50 most frequently administered agents across all hospitalization episodes. Inner rings represent major therapeutic categories, and outer rings detail more specific drug subclasses or types.

Following the procedure outlined in Figure 1A (Step 1), all EMRs for these patients were consolidated from the hospital’s information system, regardless of the reason for or department of admission. As shown in Figure 2A, the most common clinical features across all hospitalization events included Nephrotic Syndrome (*n* = 2,474 events), hyperlipidemia (*n* = 2,370), edema (*n* = 2,291), chronic kidney disease at admission (eGFR <90 mL/min/1.73 m^2^; *n* = 2,206), and hypertension (*n* = 2,157). The majority of patients were admitted under the Department of Nephrology (75.9%, 847/1095), as depicted in Figure 2B. Other contributing departments included Rheumatology and Immunology (4.93%), Endocrinology (4.12%), Emergency Medicine (3.23%), Pediatrics (2.87%), and various other clinical departments (8.96%).

To understand the therapeutic landscape, the top 50 most frequently administered medications were hierarchically classified (Figure 2C). The most frequently recorded drug classes were cardiovascular agents (e.g., antihypertensives, lipid-lowering agents, beta-blockers), anti-infectives (e.g., beta-lactams), and hematologic agents (e.g., anticoagulants and antiplatelets). Additionally, drugs for the digestive system (e.g., acid suppressants, prokinetics), endocrine and metabolic agents (e.g., antidiabetic drugs), hormones and immunomodulators (e.g., immunosuppressants, corticosteroids), and diuretics also constituted a significant proportion of the administered medications.

### Clinical features and initial medication patterns associated with hypercoagulable state

**FIGURE 3:**
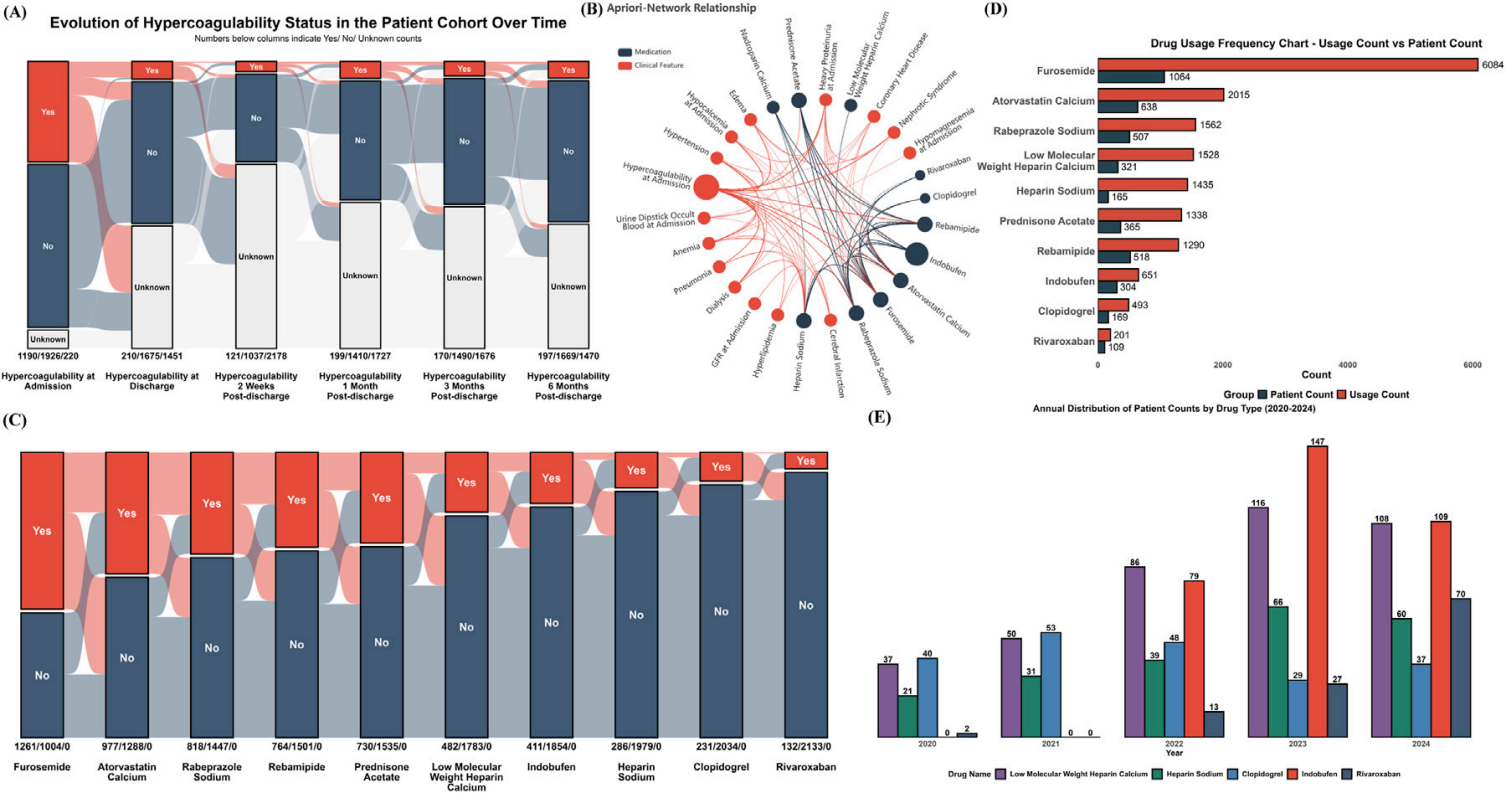
Hypercoagulable State Dynamics, Associated Factors, and Medication Use **(A)** Alluvial plot depicting the temporal evolution of hypercoagulable status from admission through 6 months post-discharge; **(B)** Apriori association network illustrating co-occurrence relationships between common clinical characteristics (red nodes) and frequently prescribed medications (blue nodes); **(C)** Yearly (2020-2024) total usage and number of unique patients for all medications identified as associated with hypercoagulable states; **(D)** Yearly (2020-2024) patient count distribution for selected anticoagulant and antiplatelet medications; **(E)** Alluvial plot illustrating co-prescription patterns in the focused cohort (*n* = 2,265 hospitalization events). Each axis represents a medication, segmented by use (Yes) or non-use (No). Flows indicate the number of events sharing specific medication combinations.

The temporal dynamics of the hypercoagulable state were assessed on a per-event basis, as illustrated in Figure 3A. The proportion of events classified as a hypercoagulable state was 38.15% (*n* = 1,190/3,119) at admission; this proportion decreased to 11.14% (*n* = 210/1,185) at discharge. Follow-up assessments showed the proportion to be 10.45% (*n* = 120/1,158) at 2 weeks, 12.37% (*n* = 199/1,609) at 1 month, 10.24% (*n* = 170/1,660) at 3 months, and 10.56% (*n* = 197/1,866) at 6 months post-discharge.

To identify clinical factors and medications robustly associated with a hypercoagulable state, an association rule analysis using the Apriori algorithm was performed on the dataset. The results, centered on the hypercoagulable state, are presented as a network in Figure 3B. This network illustrates the associations between clinical factors and medications frequently co-occurring with or used to manage this state, including various anticoagulants and antiplatelets. Subsequently, the annual total usage and the number of unique patients were tallied for medications identified as associated with the hypercoagulable state by the Apriori analysis (Figure 3C). For instance, furosemide and atorvastatin calcium, while not direct antithrombotic agents, were frequently co-prescribed in patients with hypercoagulable risk profiles and demonstrated high overall usage and patient counts.

Further analysis focused on the 5-year usage trends of specific anticoagulant and antiplatelet agents identified by the Apriori algorithm (Figure 3D). Notably, the use of indobufen and rivaroxaban increased rapidly following their clinical introduction in 2022. The use of clopidogrel showed a declining trend.

To transition from this broad exploration to a focused analysis of oral antithrombotic drug selection patterns, a specific cohort was defined for all subsequent analyses. Based on the strict inclusion and exclusion criteria detailed in the Methods section (focusing on patients who received antithrombotic therapy for a clear clinical indication). Figure 3E illustrates the intricate co-prescription patterns among core medications within this focused cohort, and Table 1 details the corresponding distributions of total dosage and duration of use for these medications.

**TABLE 1:** Characteristics of medication use in the focused cohort.

Medication	*n*	Total dose mean ± SD	Total dose median (IQR)	Unit	Duration of use, days (mean ± SD)	Duration of use, days median (IQR)
Furosemide	1261	41.9 ± 33.2	40 (20, 40)	mg	3 ± 4	1 (1, 4)
Atorvastatin calcium	977	193.5 ± 144.4	160 (80, 280)	mg	10 ± 7	9 (4, 14)
Rabeprazole sodium	818	183.5 ± 162.4	140 (20, 280)	mg	9 ± 8	7 (1, 14)
Rebamipide	764	2.5 ± 2	2.1 (0.6, 4.8)	g	9 ± 8	7 (3, 16)
Prednisone acetate	730	340.5 ± 339.2	180 (50, 500)	mg	12 ± 13	8 (3, 17)
LMWHC	482	24310 ± 27100	15375 (8000, 30000)	IU	5 ± 5	4 (2, 6)
Indobufen	411	2300 ± 1400	2800 (1200, 2800)	mg	11 ± 7	14 (6, 14)
Heparin sodium	286	54285 ± 50965	37500 (25000, 75000)	IU	4 ± 5	3 (2, 5)
Clopidogrel	231	651.6 ± 535.9	600 (225, 1050)	mg	9 ± 7	8 (3, 14)
Rivaroxaban	132	160 ± 129.5	140 (45, 210)	mg	13 ± 10	14 (3, 15)

Data are presented as n, mean ± standard deviation, or median (interquartile range). The analysis was based on a total of 2,265 hospitalization events. The column ‘n' represents the number of hospitalization events in which each specific medication was administered. ‘Total Dose’ and ‘Duration of Use’ refer to the cumulative dose and duration of therapy per hospitalization event, respectively.

Abbreviations: LMWHC, Low-Molecular-Weight Heparin; IU, International Units.

### Analysis of selection patterns for oral antithrombotic agents

**FIGURE 4:**
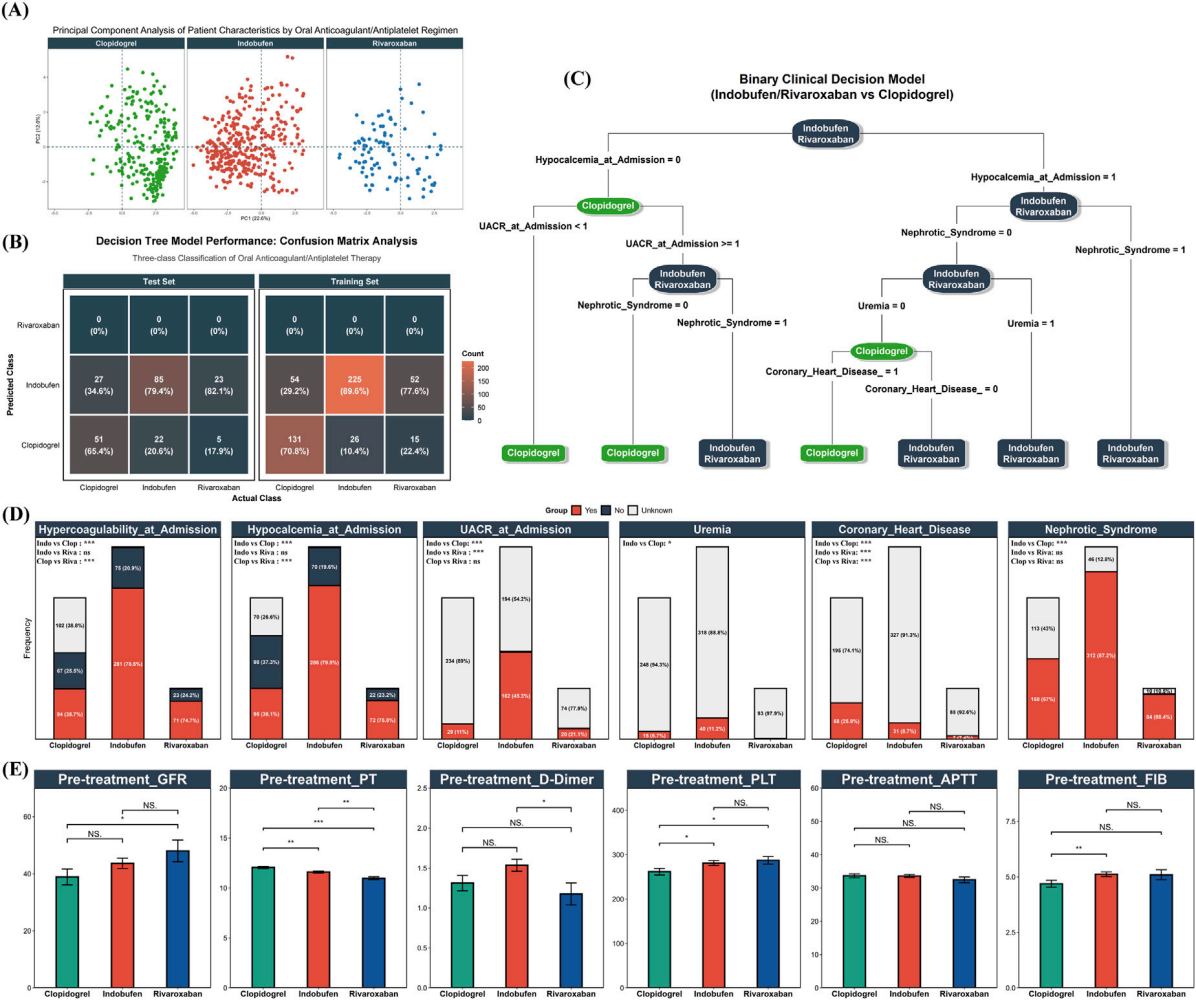
Baseline Characteristics and Oral Antithrombotic Selection. **(A)** PCA plot of 17 selected baseline clinical variables for patients receiving Clopidogrel (green), Indobufen (red), or Rivaroxaban (blue); **(B)** Confusion matrix illustrating the performance of an initial three-class decision tree model on both training and test sets; **(C)** Optimized binary clinical decision tree model distinguishing Clopidogrel use from combined Indobufen/Rivaroxaban use. Model performance: F1 score = 0.794, AUC = 0.811; **(D)** Distribution of key decision tree predictor variables across the three original drug groups; **(E)** Baseline pre-treatment levels of eGFR, PT, APTT, FIB, D-Dimer, and PLT across the three drug groups.

Differential analysis of 136 baseline variables identified 62 with significant differences between the drug groups (FDR *P* < 0.05). Beyond identifying these baseline differences, this comprehensive variable set also served to systematically screen for potential off-target adverse events. To this end, a subset of these indicators reflecting key organ function (e.g., eGFR, creatinine, hemoglobin) was tracked longitudinally at admission, discharge, and multiple post-discharge time points (2 weeks, 1, 3, and 6 months). This approach was based on the rationale that clinically significant adverse reactions would likely be captured during follow-up, either through detectable laboratory abnormalities or via their documentation in medical records. A complete comparison of all variables is provided in Supplementary Table S1. LASSO regression was then employed to refine this set to 17 key predictive variables (Table 2), which formed the foundation for the primary analysis. Following multiple imputation of missing values, Principal Component Analysis (PCA) was applied to these 17 variables to visualize the overall distribution of pre-treatment clinical profiles for each drug group (Figure 4A). The analysis revealed a substantial overlap in the multidimensional space between patients treated with indobufen and those treated with rivaroxaban, with their respective clusters located in close proximity. In contrast, patients receiving clopidogrel formed a distinct cluster, clearly separated from the other two groups.

**TABLE 2:** Comparison of key baseline variables selected by LASSO regression.

Characteristic	Rivaroxaban (*N* = 132)	Indobufen (*N* = 411)	Clopidogrel (*N* = 231)
Age, years	47.0 (28.0, 66.5)	42.5 (19.0, 60.0)	57.0 (41.0, 66.0)
Gender, n (%)			
Female	59 (62.1)	244 (68.2)	144 (54.8)
Male	36 (37.9)	114 (31.8)	119 (45.2)
Hypocalcemia at admission, n (%)	72 (75.8)	286 (79.9)	95 (36.1)
Heavy proteinuria at admission, n (%)	54 (56.8)	198 (55.3)	34 (12.9)
Hypercoagulability at admission, n (%)	71 (74.7)	281 (78.5)	94 (35.7)
Nephrotic syndrome, n (%)	84 (88.4)	312 (87.2)	150 (57.0)
Coronary heart disease, n (%)	7 (7.4)	31 (8.7)	68 (25.9)
Urine dipstick protein at admission, n (%)			
Negative	10 (10.3)	7 (2.8)	23 (32.9)
Trace	1 (1.0)	3 (1.2)	1 (1.4)
Positive	3 (3.1)	12 (4.8)	7 (10.0)
Strong positive	83 (85.6)	227 (91.2)	39 (55.7)
Urine dipstick occult blood, n (%)			
Negative	23 (23.7)	35 (13.9)	40 (41.2)
Trace	14 (14.4)	44 (17.5)	31 (32.0)
Positive	27 (27.8)	77 (30.6)	14 (14.4)
Strong positive	33 (34.0)	96 (38.1)	12 (12.4)
Total cholesterol at admission, n (%)			
ormal	23 (20.9)	65 (21.2)	60 (42.3)
Mildly elevated	14 (12.7)	38 (12.4)	27 (19.0)
Markedly elevated	73 (66.4)	203 (66.3)	55 (38.7)
Uremia, n (%)	0 (0.0)	40 (11.2)	15 (5.7)
Phospholipase A2 receptor, n (%)	14 (14.7)	33 (9.2)	7 (2.7)
Pneumonia, n (%)	59 (62.1)	271 (75.7)	216 (82.1)
Facial edema, n (%)	18 (18.9)	115 (32.1)	51 (19.4)
Diabetes mellitus, n (%)	16 (16.8)	52 (14.5)	69 (26.2)
Serum sodium, mmol/L	141.0 (139.0, 143.0)	140.0 (137.5, 142.0)	141.4 (138.8, 143.0)
Bronchitis, n (%)	21 (22.1)	49 (13.7)	66 (25.1)
Triglycerides at admission, n (%)			
Normal	44 (40.0)	116 (37.9)	79 (55.6)
Mildly elevated	19 (17.3)	58 (19.0)	22 (15.5)
Moderately elevated	40 (36.4)	118 (38.6)	38 (26.8)
Severely elevated	7 (6.4)	14 (4.6)	3 (2.1)
PDW, fL	12.2 (9.9, 15.8)	10.4 (8.9, 12.8)	10.6 (9.8, 12.2)

This table displays key demographics and 17 predictive variables identified via LASSO, regression from an initial pool of 62 significant predictors (derived from a univariate analysis of 136 total variables; see Supplementary Table S1 for full details).

Abbreviations: PDW, platelet distribution width; PLA2R, Phospholipase A2 receptor.

To further quantify and identify the clinical feature combinations that distinguish the use of these three oral agents, a decision tree model was developed based on the 17 selected variables. A preliminary three-class model designed to differentiate among all three groups was evaluated. The confusion matrices for the training and test sets (Figure 4B) demonstrated that the model had very limited ability to distinguish between indobufen and rivaroxaban, a finding consistent with the high degree of similarity observed in the PCA. Given the high degree of similarity in baseline features and the initial model’s performance, the indobufen and rivaroxaban groups were combined into a single “Indobufen/Rivaroxaban” group. A binary decision tree analysis was then conducted to distinguish this combined group from the “Clopidogrel” group. The final model demonstrated strong discriminatory performance, achieving an F1 score of 0.794 and an AUC of 0.811. The structure of the final decision tree is presented in Figure 4C, highlighting the key decision nodes: Hypocalcemia at Admission, Urine Albumin-to-Creatinine Ratio (UACR) at Admission, NS, Uremia, and Coronary Heart Disease. To visually illustrate the differences in these key predictors, Figure 4D displays their distributions across the original three drug groups, along with the results of pairwise statistical comparisons. Finally, Figure 4E presents the baseline levels of a key renal function indicator (eGFR) and coagulation-related parameters (PT, APTT, FIB, D-Dimer, PLT) across the three groups. Notably, despite rivaroxaban being an anticoagulant and indobufen an antiplatelet agent, there were no clinically significant differences in their pre-treatment PLT or coagulation function indicators.

### Comparative efficacy and safety of rivaroxaban and indobufen

**FIGURE 5:**
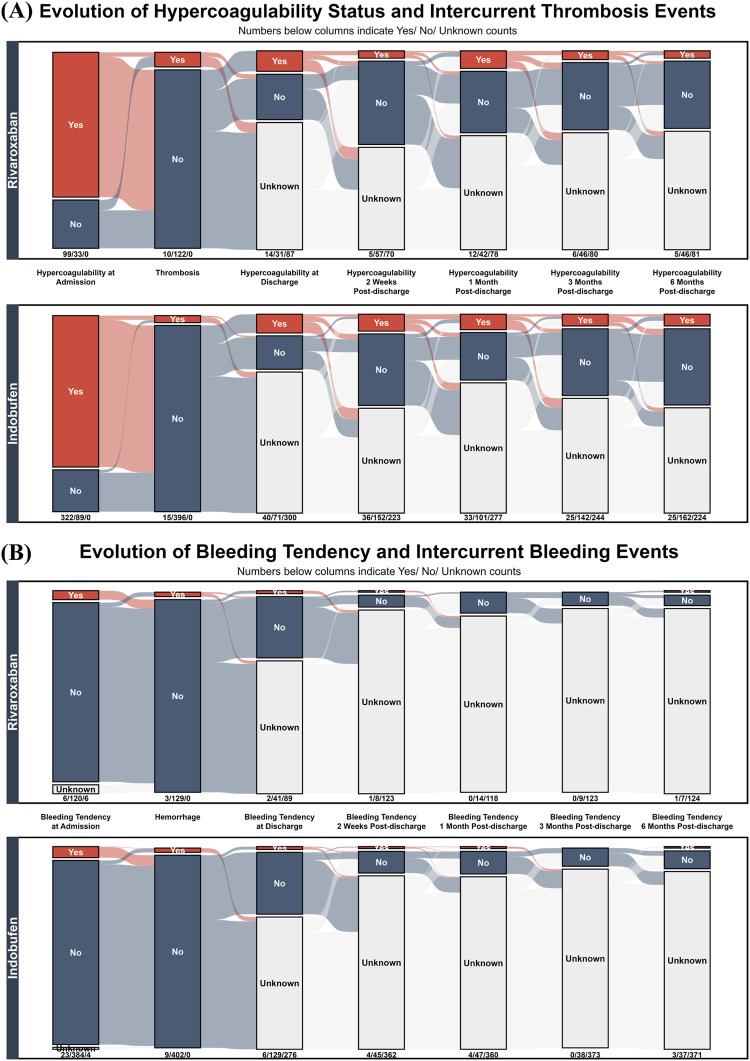
Longitudinal Trajectories of Thrombotic and Bleeding Events in Rivaroxaban and Indobufen Cohorts. Alluvial plots showing the temporal evolution of **(A)** hypercoagulability/thrombosis and **(B)** bleeding tendency/hemorrhage in patients treated with Rivaroxaban (top panels) versus Indobufen (bottom panels). The plots track patient status from admission to 6 month follow-up.

Having established baseline comparability, we directly compared the longitudinal efficacy and safety of the two drugs. Figure 5A tracks the evolution of hypercoagulable states and the incidence of in-hospital thrombotic events. At admission, both treatment groups exhibited a similarly high prevalence of hypercoagulability: 75.00% (99/132 events) in the rivaroxaban group and 78.35% (322/411 events) in the indobufen group. The incidence of new-onset thrombotic events during hospitalization was 7.58% (10/132 events) for the rivaroxaban group and 3.65% (15/411 events) for the indobufen group (Chi-squared test, *P* = 0.101). Among the subgroup of events with pre-existing hypercoagulability at admission, the incidence of in-hospital thrombosis was 3.03% (3/99 events) for rivaroxaban and 2.80% (9/322 events) for indobufen (Fisher’s exact test, *P* = 1). Following in-hospital treatment, the prevalence of hypercoagulability decreased significantly in both groups at discharge. This resolved state was largely sustained in both cohorts throughout the 6 month follow-up period.

Complementing the efficacy analysis, Figure 5B presents a safety comparison by illustrating the longitudinal evolution of bleeding tendency and the incidence of in-hospital hemorrhage. At admission, both cohorts exhibited a similarly low prevalence of bleeding tendency: 4.55% (6/132 events) for the rivaroxaban group and 5.60% (23/411 events) for the indobufen group (Chi-squared test, *P* = 0.879), indicating comparable baseline safety profiles. The incidence of in-hospital hemorrhage events was also nearly identical and remained low in both groups: The incidence of in-hospital hemorrhage events was also low and statistically indistinguishable between the groups: 2.27% (3/132 events) for rivaroxaban and 2.19% (9/411 events) for indobufen (Fisher’s exact test, *P* = 1).

Table 3 details the distribution of thrombotic events, hemorrhage events, hypercoagulability at discharge, and bleeding tendency at discharge across different dosing regimens of rivaroxaban and indobufen. Notably, the incidence of hypercoagulability at discharge in the indobufen 10 mg bid group (13.27%, 28/211) was significantly higher than in the 20 mg qd group (3.05%, 4/131). Compared to the 10 mg bid regimen, the 20 mg qd regimen was associated with a lower likelihood of persistent hypercoagulability at discharge (OR = 0.19; 95% CI, 0.04 - 0.64; *P* = 0.004).

**TABLE 3:** Distribution of thrombotic and bleeding outcomes by dosing regimen.

Drug	n	Thrombotic events	Hypercoagulability at discharge	Hemorrhage events	Bleeding tendency at discharge
Rivaroxaban					
10 mg, qd	70	3 (4.29%)	7 (10%)	1 (1.43%)	1 (1.43%)
15 mg, qd	53	2 (3.77%)	5 (9.43%)	1 (1.89%)	1 (1.89%)
20 mg, qd	4	2 (50%)	1 (25%)	0 (0%)	0 (0%)
10 mg, bid	3	2 (66.67%)	1 (33.33%)	1 (33.33%)	0 (0%)
15 mg, bid	2	1 (50%)	0 (0%)	0 (0%)	0 (0%)
Indobufen					
100 mg, qd	45	2 (4.44%)	5 (11.11%)	4 (8.88%)	0 (0%)
200 mg, qd	131	6 (4.58%)	4 (3.05%)	1 (0.76%)	1 (0.76%)
100 mg, bid	211	7 (3.32%)	28 (13.27%)	4 (1.9%)	4 (1.9%)
200 mg, bid	24	0 (0%)	2 (8.33%)	0 (0%)	1 (4.17%)

Due to the small number of hospitalizations in some groups, no statistical comparisons were made.

Abbreviations: qd, once daily; bid, twice daily.

It is noteworthy that of the 12 total in-hospital hemorrhage events, 11 (91.67%) occurred in patients assessed as having ‘no’ bleeding tendency at admission. This suggests that the currently used criteria for assessing bleeding tendency may be insufficient for predicting clinical hemorrhage, and future work could focus on developing more suitable predictive models based on actual bleeding outcomes.

### Analysis of effects of target oral antithrombotic agents on laboratory

Before assessing treatment effects, we first confirmed that the treatment exposure was comparable between the two drug cohorts. Table 1 demonstrates similar durations of use for indobufen and rivaroxaban, which provides the rationale for a direct pre-post comparison. Therefore, paired t-tests were initially used to quantify the changes in various laboratory parameters from pre-to post-treatment. Due to the large number of parameters with significant changes, Table 4 focuses on presenting a selection of the most clinically relevant and statistically significant results. A complete analysis of all tested variables can be found in Supplementary Table S2.

**TABLE 4:** Comparison of changes in laboratory parameters before and after treatment.

Characteristic	Rivaroxaban				Indobufen			
	*n*	Baseline mean	Follow-up mean	*P*-value	n	Baseline mean	Follow-up mean	*P*-value
Part A: Convergent effects								
Nutrition/Inflammation								
Prealbumin	65	185.93	327.37	<0.001	176	216.15	353.88	<0.001
Albumin	68	18.15	24.94	<0.001	179	20.37	26.5	<0.001
WBC	66	10.42	12.97	<0.001	189	9.83	12.38	<0.001
Renal function								
Urine protein	28	2.57	1.23	<0.001	78	2.31	1.02	<0.001
Urine occult blood	36	1.35	0.77	0.001	104	1.29	0.44	<0.001
Serum calcium	69	1.88	2.05	<0.001	204	1.92	2.1	<0.001
Lipid profile								
LDL-C	48	6.84	5.39	<0.001	135	6.24	4.56	<0.001
HDL-C	48	1.42	1.98	<0.001	133	1.48	2.06	<0.001
Hemostasis/Coagulation								
MPV	55	9.4	8.95	<0.001	181	9.45	8.96	<0.001
PDW	61	12.72	14.22	0.003	179	11.05	13.64	<0.001
FIB	20	5.42	3.95	0.008	62	5.24	4.19	0.001
Antithrombin III	9	55.88	87.18	0.026	38	74.64	90.29	0.004
Part B: Rivaroxaban’s pharmacological fingerprint								
D-dimer	17	3.26	2.2	0.043	51	2.36	3.22	0.101
Creatinine	70	134.9	117.58	0.035	199	175.9	179.28	0.711
TBA	68	2.62	4.43	<0.001	181	4.48	5.54	0.263
Ig G	38	4.08	5.05	0.002	71	5.42	5.63	0.527
Cystatin C	44	1.83	1.62	0.046	171	2.09	2.19	0.11
Part C: Indobufen’s pharmacological fingerprint								
APTT	20	35.16	36.45	0.548	62	35.92	32.23	0.011
Plateletcrit	58	0.29	0.28	0.745	177	0.28	0.26	0.002
ApoB	21	1.94	1.61	0.077	103	1.66	1.31	<0.001
HGB	65	121.34	123.8	0.21	182	121.36	124.85	0.004
GGT	67	55.02	52.14	0.652	180	45.98	59.24	<0.001
Part D: Divergent effects								
eGFR	44	43.32	49.28	0.014	172	46.1	42.76	0.016

Abbreviations: ApoB, Apolipoprotein B; eGFR, estimated glomerular filtration rate; FIB, fibrinogen; HGB, hemoglobin; HDL-C, high-density lipoprotein cholesterol; Ig G, Immunoglobulin G; LDL-C, low-density lipoprotein cholesterol; MPV, mean platelet volume; WBC, white blood cell count; GGT, Gamma-Glutamyl Transferase; TBA, total bile acid.

**FIGURE 6:**
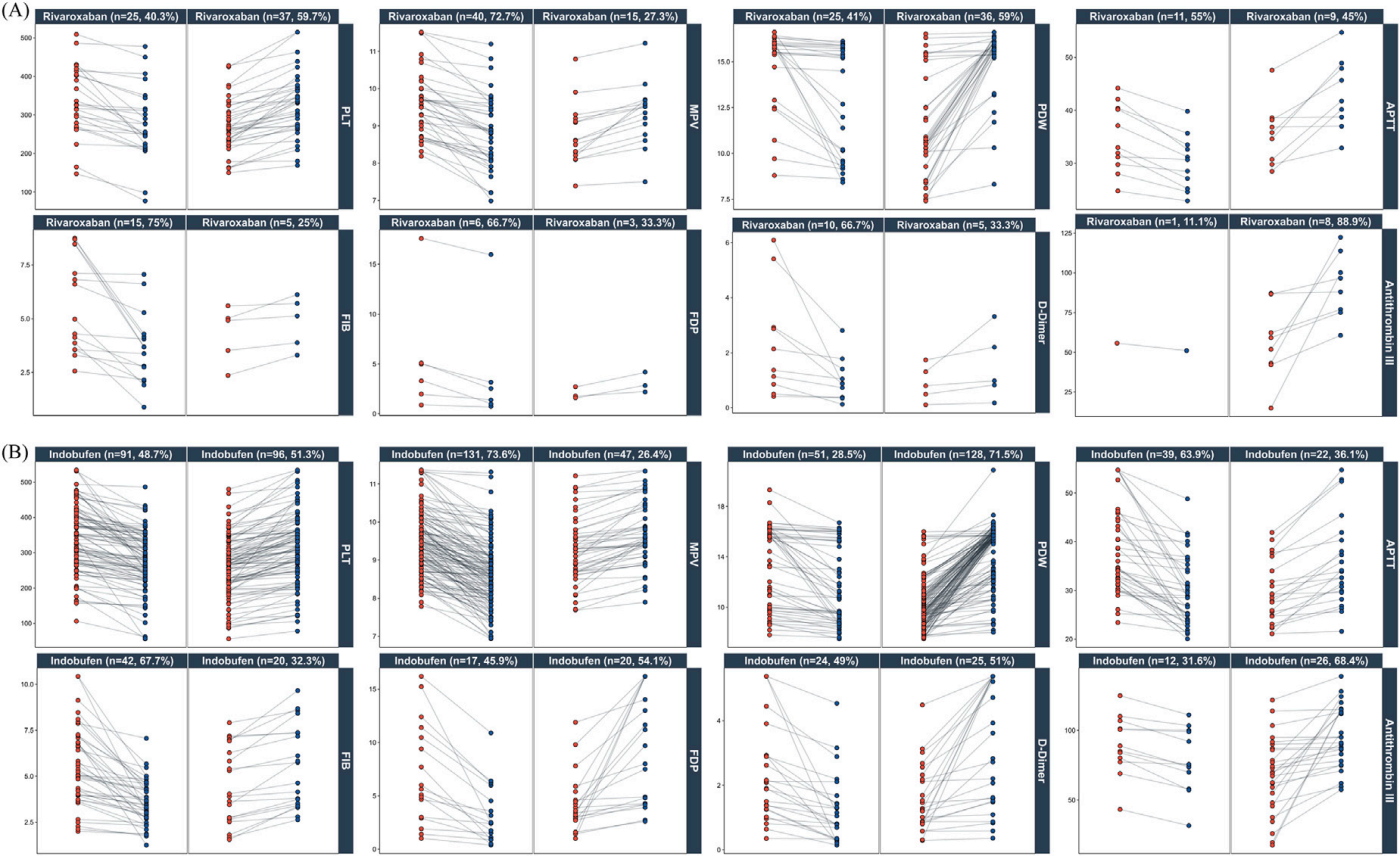
Individual patient responses to Rivaroxaban and Indobufen. Paired plots show changes in key platelet and coagulation parameters from pre-treatment (red) to post-treatment (blue). Panels are stratified by drug (**(A)**: Rivaroxaban; **(B)** Indobufen) and further subdivided by the direction of change (decrease vs. increase). Percentages in the panel titles indicate the proportion of patients in each subgroup.

Recognizing that aggregate statistics such as mean differences can mask inter-individual heterogeneity, we used paired plots to visualize the trajectories of individual patient parameters. In the rivaroxaban group (Figure 6A), a decreasing trend was observed for parameters including MPV (72.7%), FIB (75.0%), FDP (77.8%), D-Dimer (66.7%), and APTT (65.0%). Conversely, PDW (59.0%) and Antithrombin III (88.9%) showed an increasing trend. In the indobufen group (Figure 6B), MPV (73.6%) and FIB (67.7%) showed a decreasing trend, while PDW (71.5%), APTT (63.9%), and Antithrombin III (68.4%) showed an increasing trend.

Although paired t-tests revealed significant improvements in the lipid profile (e.g., LDL-C, HDL-C), these non-hemostatic changes were excluded from detailed visualization. These effects are more plausibly attributed to confounding by widespread concomitant statin therapy (Figure 3) rather than a direct pharmacological effect of the study drugs, for which there is limited mechanistic evidence. To further control for confounding factors and quantify the independent effects of the drugs, we developed a LMM. This model included age, sex and major co-medications as fixed effects, with individual hospitalization events treated as a random effect. The model results (Table 5; Supplementary Table S3) provide an assessment of the main drug effect (baseline differences between groups) and the interaction effect (the drug’s independent influence on the parameter’s trajectory).

**TABLE 5:** Independent effects of indobufen on laboratory parameters identified by linear mixed-effects model.

Characteristic	No. of events, N (*n*)	Main effect (baseline)		Interaction effect (trend)	
		Estimate	*P*-value	Estimate	*P*-value
Part A: Independent effects with comparable baselines					
MPV	464 (174)	−0.10	0.335	−0.32	<0.001
PDW	464 (174)	−0.24	0.441	0.95	0.006
Plateletcrit	464 (174)	0.01	0.41	−0.02	0.006
Prealbumin	469 (167)	−21.56	0.082	56	<0.001
CRP	223 (48)	−6.93	0.311	19.37	0.002
Urine occult blood	409 (142)	0.08	0.451	−0.37	<0.001
HDL-C	388 (135)	−0.01	0.876	0.17	0.033
FT4	212 (49)	−1.63	0.054	3.13	<0.001
T3	212 (49)	−0.14	0.113	0.17	0.01
LAP	469 (167)	1.16	0.599	4.8	<0.001
Part B: Independent effects with significant baseline differences					
Globulin	474 (170)	−3	<0.001	2.32	<0.001
Total protein	476 (172)	−6.11	<0.001	4.06	0.001
Albumin	479 (171)	−3.7	<0.001	2.26	0.003
Urine dipstick protein	374 (137)	0.52	<0.001	−0.39	<0.001
Urine protein	383 (96)	2.13	0.023	−1.87	0.013
Serum calcium	525 (193)	−0.06	0.004	0.05	0.021
Uric acid	516 (193)	−29.74	0.035	27.45	0.043
Serum bicarbonate	518 (193)	1.35	0.003	−1.49	0.001
LDL-C	388 (135)	0.82	0.01	−0.91	0.001
Total cholesterol	396 (140)	1.29	0.001	−0.99	0.001
ApoB	333 (106)	0.19	0.021	−0.17	0.011
Lipoprotein a	325 (102)	185.7	0.046	−168.01	0.022
T4	212 (49)	−14.08	0.003	16.51	<0.001
CHE	474 (170)	1229.94	<0.001	−656.47	0.001
Part C: Associations No longer significant after confounder adjustment					
eGFR	428 (166)	−1.02	0.718	−0.56	0.756
HGB	475 (175)	8.27	0.004	0.128	0.939
Hematocrit	475 (175)	2.31	0.006	−0.05	0.919
APTT	237 (71)	2.04	0.235	−1.83	0.29
FIB	237 (71)	0.79	0.011	−0.30	0.249

“*N*” represents the total number of events (indobufen group and control group) included in the model; “*n*” represents the number of events from the indobufen group. The model was adjusted for age, sex, and major concomitant medications (Figure 3), with patient included as a random effect. Main Effect: Assesses the baseline difference between the indobufen and control groups. Interaction Effect: Quantifies the independent influence of indobufen on the parameter’s change over time. For complete model outputs, including estimates for all covariates, see Supplementary Table S3.

Abbreviations: LAP, leucine aminopeptidase; CHE, cholinesterase.

Using MPV as an example, the model showed no significant difference in baseline levels between the indobufen group and the non-user group (main drug effect: P = 0.335), indicating good comparability between the groups. However, the model revealed a significant negative interaction effect (Estimate = −0.32, *P* = 0.0003), indicating that after controlling for multiple confounders, indobufen use was independently associated with a more substantial decrease in MPV. These independent pharmacological associations included significant decreases in MPV and plateletcrit, and significant increases in PDW, prealbumin, CRP, and HDL-C (all interaction effects *P* < 0.05).

In contrast, for LDL-C, the model showed that the indobufen group had significantly higher baseline levels (main drug effect: *P* = 0.0097), suggesting a baseline imbalance. Although a significant interaction effect was observed (*P* = 0.0005), its attribution is complex due to the baseline differences and widespread co-administration of lipid-lowering drugs. The attribution of this effect is complex, potentially stemming from three non-mutually exclusive factors: (1) a form of confounding by severity, where patients with markedly higher baseline levels likely received more aggressive, unmodeled lipid-lowering therapy, leading to a greater observed reduction; (2) a synergistic interaction with concomitant medications such as atorvastatin; or (3) a genuine, independent lipid-modulating effect of indobufen itself. Differentiating between these possibilities is beyond the scope of this retrospective analysis and awaits confirmation from future prospective and mechanistic studies.

## Discussion

The premise of our study is not a pharmacological hypothesis but an empirical observation of “*de facto* therapeutic interchangeability.” This observation stemmed from our initial finding that indobufen and rivaroxaban have become the primary emerging oral antithrombotic choices in recent years. Subsequent analysis then revealed that clinicians are applying these two drugs to a highly similar patient phenotype, particularly those with NS, while reserving agents like clopidogrel for distinct indications. This distinction is critical, as a direct comparison with clopidogrel would have been invalidated by severe confounding by indication, creating a methodological paradox where a seemingly appropriate peer-drug comparison would actually involve two fundamentally different patient populations with insufficient overlap in their baseline characteristics. In contrast, the observed interchangeability between indobufen and rivaroxaban presented a unique opportunity to address a zone of genuine clinical equipoise. Our study was therefore designed not to compare mechanisms in a vacuum, but to resolve a real-world clinical dilemma: in a population where clinicians already treat these two drugs as viable alternatives, which strategy offers a better net clinical benefit? This spontaneous adoption of functionally equivalent options, driven by real-world complexity, is a common feature of clinical practice when managing frail, comorbid, or high-risk patients (Campitelli et al., 2021; Mohamed et al., 2021; Gorog et al., 2023), often requiring individualized adjustments beyond standard guidelines.

Regarding the primary endpoints (Figure 5), indobufen showed a numerically lower rate of thrombosis compared to rivaroxaban (3.65% vs. 7.58%). While not statistically significant, this trend suggests a potential thromboprotective advantage for indobufen that warrants investigation in larger RCTs. Crucially, this potential benefit did not come at the cost of safety, as in-hospital bleeding rates were low and comparable between the groups (2.19% vs. 2.27%). This balanced profile is of major clinical importance for CKD patients, who must navigate high risks of both thrombosis and bleeding. As for secondary outcomes, both treatments effectively mitigated the laboratory-defined “hypercoagulable state” through 6 months of follow-up and performed similarly well in the highest-risk subgroup of patients with pre-existing hypercoagulability. It is crucial to acknowledge the significant heterogeneity of the “hypercoagulable state” as a diagnostic label. Its underlying mechanisms are disease-specific, and its diagnostic criteria vary widely across conditions without a universal gold standard (Johansson et al., 2022; Marchetti et al., 2022; Song et al., 2022; Lim et al., 2023). Moreover, composite biomarkers often fail to capture the true coagulation status (Scheiner et al., 2022; Lim et al., 2023). To address this ambiguity, our study bypassed the composite label and instead analyzed the core laboratory parameters independently. Since these individual markers are standardized and widely applied, this approach provides objective, granular data to support individualized assessments that can be adapted to the diverse diagnostic criteria used in practice.

Our analysis of hemostatic markers revealed a dual pattern: convergent effects reflecting overall clinical improvement and distinct pharmacological fingerprints unique to each drug. The convergent effects included mitigation of the procoagulant environment in both groups, evidenced by decreased FIB and MPV, and increased antithrombin III. The fingerprints themselves were perfectly aligned with each drug’s mechanism: Rivaroxaban’s pharmacological fingerprint was characterized by a marked suppression of D-Dimer, indicating reduced systemic coagulation, whereas Indobufen’s pharmacological fingerprint was more pronounced in its impact on platelet-related indices like plateletcrit. However, despite these clear group-level profiles, the significant inter-individual variability shown in Figure 6 highlights the limitations of simple pre-post comparisons.

Moreover, in clinical practice, patients often receive combination therapy, creating a significant risk of confounding bias where observed changes cannot be attributed to indobufen alone. To isolate the effects of indobufen from the confounding bias of combination therapy, we employed a LMM, adjusting for age, sex, and key concomitant medications. This analysis provided more robust evidence for indobufen’s known antiplatelet activity (Liu et al., 2023; Dai C. et al., 2024; Dai W.-B. et al., 2024), Reinforcing its independent role in decreasing MPV and plateletcrit while increasing PDW (Table 5, Part A). More interestingly, the LMM uncovered potential pleiotropic effects, linking indobufen independently to increases in prealbumin and HDL-C. This finding is supported by recent research suggesting indobufen can modulate the MAPK inflammatory pathway (Bai et al., 2024). However, this pleiotropy has clear boundaries, it does not extend to broad antioxidant effects, as indobufen does not alter F2-isoprostane generation driven by oxidative stress (Davì et al., 1999). Finally, while the model also linked indobufen to elevated thyroid hormones (FT4, T3), we interpret this not as a direct drug effect but as a likely confounding signal. Such fluctuations are characteristic of Non-Thyroidal Illness Syndrome (NTIS) in CKD patients and probably reflect overall clinical improvement rather than a specific pharmacological action (Yan et al., 2017; Mahashabde et al., 2024).

A key advantage of the LMM approach is its ability to disentangle initial findings from the effects of confounding. This is best illustrated by the hemoglobin result. Initially, the significant rise in HGB in the indobufen group was a particularly noteworthy finding, supporting the hypothesis that its reversible COX-1 inhibition confers superior gastrointestinal safety (Dai W.-B. et al., 2024; Ren et al., 2024). However, this effect became non-significant in the LMM after adjusting for co-medications. This highly informative “negative” result indicates the improvement was likely due to co-prescribed PPIs or mucosal protectants, not indobufen itself. Likewise, the concerning decline in eGFR seen in the initial analysis, which raised fears of nephrotoxicity (Hanaoka et al., 2025), also became non-significant in the LMM. This suggests the effect was likely confounded by co-administered glucocorticoids rather than being a direct drug-induced toxicity. Ultimately, the LMM served a crucial dual purpose: it isolated indobufen’s true independent effects while preventing the misattribution of effects from other drugs—be they protective (PPIs) or potentially harmful (steroids). This highlights a fundamental principle for real-world data analysis: traditional comparisons are inadequate for handling the complex, time-varying confounders inherent in clinical practice. Advanced longitudinal models are therefore essential to disentangle these factors, convert messy clinical reality into clear insights, and accurately isolate the independent effect of a drug. Our findings caution clinicians against prematurely attributing changes in hemoglobin or eGFR to indobufen itself, highlighting the need to consider the confounding effects of co-prescribed PPIs and steroids.

Finally, this study has several limitations that inform future research. Our analysis framework, designed to analyze real-world EHR data, cannot directly measure pharmacokinetic (PK) or pharmacodynamic (PD) parameters. Its utility lies in generating hypotheses from large-scale clinical observations. For instance, the analysis was structured to compare drug-level effects rather than dose-specific outcomes—a methodological choice to avoid the model overfitting and loss of statistical power that would result from fragmenting the cohort into smaller subgroups. This approach revealed that patients receiving indobufen “100 mg, bid” showed a significantly greater resolution of hypercoagulability than those on “200 mg, qd,” despite an identical total daily dose (Table 3). This finding suggests that dosing frequency, and by extension the underlying PK/PD profile, may influence clinical efficacy. It raises a question that our RWD platform is suited to identify but not directly answer: why the same daily dose yields different outcomes. Answering this requires a dedicated mechanistic study integrating our findings with direct PK/PD measurements (e.g., drug concentrations via LC-MS and platelet function via thromboelastography). Therefore, this study serves not as a final conclusion, but as a foundational step to guide future, targeted mechanistic research.

## Data Availability

The raw data supporting the conclusions of this article contain sensitive patient information and are not publicly available due to privacy regulations and restrictions imposed by the institutional ethics committee of The First People’s Hospital of Qinzhou. However, a de-identified and interactive visualization of the dataset is accessible through a web application, which allows for the exploration of data distributions and key clinical characteristics presented in this study. Further inquiries or requests for access to aggregated, non-identifiable data can be directed to the corresponding author.

## Abbreviations

APTT, Activated Partial Thromboplastin Time; CKD, Chronic Kidney Disease; DOAC, Direct Oral Anticoagulant; eGFR, estimated Glomerular Filtration Rate; EMRs, electronic medical records; FIB, Fibrinogen; HIS, Hospital Information System; LASSO, Least Absolute Shrinkage and Selection Operator; LIS, Laboratory Information System; LLM, Large Language Model; LMM, Linear Mixed Model; LMWH, Low-Molecular-Weight Heparin; PCA, Principal Component Analysis; PLT, Platelet Count; PT, Prothrombin Time; PK, Pharmacokinetic; PD, pharmacodynamic.
