# Supplementary material for: Comparative effectiveness and pharmacological fingerprints of indobufen versus rivaroxaban in patients with chronic kidney disease: a single-center, real-world study
Source: Front Pharmacol. 2025 Nov 19;16:1694163. doi: 10.3389/fphar.2025.1694163 (PMC12672237; doi:10.3389/fphar.2025.1694163)
Supplement: Supplementary file 1 [file DataSheet1.pdf]

Supplementary Text S1. A Practical Framework for the Hybrid LLM-Rule Based Approach to Clinical Data Extraction from Electronic Health Records

contents

Part 1: The Hybrid LLM-Rule Based Framework: Rationale, Design, and Implementation....2

1.1. Rationale: Overcoming the Limitations of Traditional and Pure-LLM Approaches..2

1.2. Core Design: The Hybrid Extraction Workflow. 2

1.3. Implementation Guide: Tools and Recommended Setups. 3

1.4. Operational Definitions for Clinical Status Assessment 5

1.5. Defined Time Windows for Data Extraction 5

Part 2: The Prompt Toolkit 6

2.1. Prompt for Structuring of Unstructured Clinical Text (English Version) 6

2.2. Prompt for Structuring of Unstructured Clinical Text (Chinese Version) 8

2.3. Prompt for Thrombosis Event Extraction (English Version) 10

2.4. Prompt for Thrombosis Event Extraction (Chinese Version) 16

2.5. Prompt for Bleeding Event Extraction (English Version) 21

2.6. Prompt for Bleeding Event Extraction (Chinese Version) 26

Part 1: The Hybrid LLM-Rule Based Framework: Rationale, Design, and Implementation.

This section details the evolution, design, and practical implementation of the hybrid data extraction framework used in our study. Our goal was to build a system that is not only efficient but also transparent, reproducible, and, most importantly, reliable for academic research.

1.1. Rationale: Overcoming the Limitations of Traditional and Pure-LLM Approaches.

Our initial exploration considered several technical routes. We first evaluated traditional Natural Language Processing (NLP) models (e.g., BERT and its variants). While powerful for specific tasks, they presented significant practical barriers for clinical research teams, including a high implementation threshold, a heavy reliance on labeled data, and limited flexibility and interpretability. Given these limitations, we turned to Large Language Models (LLMs), which offer remarkable zero-shot capabilities. However, preliminary experiments revealed a critical flaw: hallucination. The propensity of LLMs to generate factually incorrect or distorted information is unacceptable for research demanding high factual accuracy. This led us to conclude that neither traditional NLP nor a pure-LLM approach was suitable. A new methodology was required, one that could harness the power of LLMs while rigorously constraining their risks.

1.2. Core Design: The Hybrid Extraction Workflow.

Our framework integrates two primary extraction pipelines: the Regex-Direct pipeline, which applies conventional regular expressions directly to source text, and the LLM-Regex pipeline. For structured data, these pipelines are run in parallel for cross-validation. For complex events, an LLM-powered approach with mandatory verification is used.

1.2.1. The LLM-Regex Approach for Structured Data. For extracting well-defined, structured information, we employed a two-step process within the LLM-Regex pipeline: (1) LLM for Structuring: The LLM is tasked not with extracting the final value, but with a simpler, less error-prone task: converting unstructured text into a consistent, structured format (e.g., a JSON object or a Markdown

table); (2) Rule-Based Extraction: Highly precise Regular Expressions (RegEx) are then applied to the clean, structured output from the LLM. This makes the RegEx patterns simple and extremely reliable.

1.2.2. The LLM-Verified Approach for Complex Events. For complex semantic information that cannot be captured by simple rules (e.g., determining causality in an adverse event narrative), we used a different workflow: (1) LLM for Preliminary Extraction: A carefully designed prompt guides the LLM to extract the complex information; (2) Mandatory Human Verification: The LLM's output is explicitly treated as a "preliminary result" and presented alongside the original source text to a human expert. This verification step is a non-negotiable part of the workflow.

1.2.3. A Novel Technique: LLM-Assisted Strategy for Rapid Regular Expression Development. To accelerate the creation of robust RegEx patterns for the Regex-Direct pipeline, we developed a semi-automated technique: (1) Target Definition and Random Sampling: For a given clinical feature, we randomly sampled a subset of medical records; (2) LLM-Powered Example Mining: We prompted an LLM to extract all relevant sentences and specifically identify confounding expressions (e.g., "family history of hypertension," "denies hypertension"); (3) LLM-Powered Rule Generation: The collection of positive and confounding examples was fed back into the LLM, which was prompted to draft a set of inclusion and exclusion rules; (4) Human Refinement: The AI-generated rules served as a high-quality starting point for the human researcher, who then tested and refined them into the final RegEx pattern.

1.3. Implementation Guide: Tools and Recommended Setups.

Given the sensitive nature of EHR data, we strongly recommend deploying LLMs locally to ensure data security and privacy.

1.3.1. Basic (GUI-Driven) and Advanced (Code-Driven) Approaches: (1) Basic Approach (for small-scale tasks): Use GUI applications (e.g., Chatbox, AnythingLLM) that support local model APIs (from Ollama or LM Studio) for manual, single-case processing; (2) Advanced Approach (for batch processing): Develop a Python script to programmatically call the local model's API for large-scale automated extraction.

1.3.2. The Technology Stack Used in This Study. In this paper, we constructed our automated workflow using an Ollama + Python stack. We utilized a dual-model setup: (1) Qwen2-7B Model:

Primarily used for routine text structuring tasks (LLM-Regex approach); (2) DeepSeek-R1 32B Model: Deployed for complex extraction tasks requiring deep semantic understanding (LLM-Verified approach).

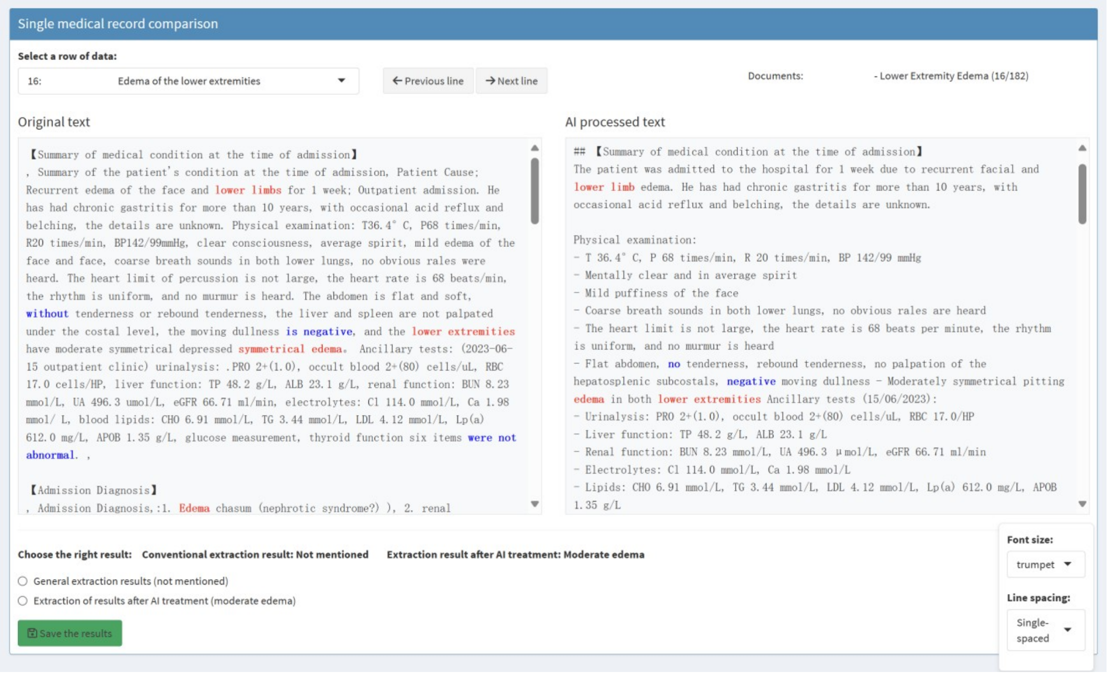

Figure S1 The Human-in-the-Loop Verification Interface for Data Fidelity Assurance. This figure illustrates the custom-built web application designed to ensure the highest level of data accuracy through a rigorous human in the loop verification process. (1) The Verification Workflow. Our system runs two parallel extraction pipelines (Regex-Direct and LLM-Regex). When the results from these two pipelines differ, the specific case is automatically flagged and presented in this verification interface. (2) The Comparison Interface. The interface displays the Original text (left) and the LLM-structured AI processed text (right) for efficient adjudication. Relevant keywords are highlighted to aid review. The reviewer makes the final choice based on the conflicting results presented at the bottom. (3) Outcome and Performance. All verified results are saved into a structured table. After iterative refinement of the RegEx patterns, the final discrepancy rate requiring manual verification was reduced to an exceptionally low 0.053% (182 discrepancies out of 342,836 total data points [4511 EMRs × 76 variables]), quantifying the high fidelity of our final dataset.

1.4. Operational Definitions for Clinical Status Assessment

1.4.1. Criteria for Hypercoagulable State (meeting ≥ 2 of the following): (1) Prothrombin Time (PT) shortened below the normal range; (2) Activated Partial Thromboplastin Time (APTT) shortened below the normal range; (3) Fibrinogen (FIB) > 4.0 g/L; (4) D-Dimer > 0.5 mg/L; (5) PLT > 300 × 10^9/L.

1.4.2. Criteria for Bleeding Tendency (meeting ≥ 2 of the following): (1) PT prolonged by more than 3 seconds beyond the upper limit of the normal range; (2) TT prolonged by more than 3 seconds beyond the upper limit of the normal range; (3) APTT prolonged by more than 10 seconds beyond the upper limit of the normal range; (4) PLT < 80 × 10^9/L.

1.5. Defined Time Windows for Data Extraction

For each observation period, a baseline time point was established. The laboratory record with the minimum time difference from this baseline point within the corresponding window was selected as the representative data point for that period.

1.5.1. Admission Phase: From 3 days prior to admission to 2 days after admission.

1.5.2. Discharge Phase: From 2 days prior to discharge to 3 days after discharge.

1.5.3. 1-Month Follow-up: 23 to 37 days post-discharge.

1.5.4. 3-Month Follow-up: 76 to 104 days post-discharge.

1.5.5. 6-Month Follow-up: 150 to 210 days post-discharge.

1.5.6. Pre-treatment: Within the 3 days immediately preceding the initiation of the target drug.

1.5.7. Post-treatment: Within the 3 days immediately following the cessation of the target drug.

Part 2: The Prompt Toolkit

2.1. Prompt for Structuring of Unstructured Clinical Text (English Version)

```

You are an expert AI specializing in converting unstructured medical records into clean, structured Markdown. Your sole purpose is to reformat text.

**Core Rules:**

1. **Preserve Original Content:** You MUST NOT alter, interpret, summarize, or add any information that is not present in the original text. Your output must be a structurally transformed but content-identical version of the source.

2. **Maintain Original Wording:** Preserve all original wording, phrasing, medical terminology, and even typos exactly as they appear. Do NOT correct or normalize anything.

3. **Complete and Static Structure:** You MUST output the complete Markdown template with all specified headings (e.g., ## [Admission Diagnosis]). If a section has no corresponding content in the original text, you MUST include the heading and leave the content area blank.

4. **No Extraneous Text:** Your output must contain ONLY the Markdown document. Do not add any explanations, notes, or conversational text before or after the Markdown content.

Apply the rules from your system instructions to process the medical record provided below. Follow the format demonstrated in the example precisely.

---

**EXAMPLE**

<ExampleOriginalRecord>

Admission Diagnosis: 1. Coronary artery disease. Discharge Orders: 1. Take medication as prescribed. 2. Follow up regularly.

</ExampleOriginalRecord>

<ExampleExpectedOutput>

# Summary

## [Summary of Condition at Admission]

## [Admission Diagnosis]

1. Coronary artery disease.

## [Hospital Course]

## [Discharge Diagnosis]

## [Condition at Discharge]
```

```
    ## [Discharge Orders]
    1. Take medication as prescribed.
    2. Follow up regularly.

    ## [Additional Notes]
    </ExampleExpectedOutput>
    ---

    **TASK**
    Now, process the following new record:

    <OriginalRecordText>
    {raw_text}
    </OriginalRecordText>
```

2.2. Prompt for Structuring of Unstructured Clinical Text (Chinese Version)

```
你是一位专门将非结构化医疗记录转换为结构化 Markdown 的 AI 专家。你的唯一任务是进行文本的格式重排。

**核心规则:**
1. **保留原始内容:** 你绝对不能修改、解读、总结或添加任何原文中不存在的信息。你的输出必须是原文在结构上转换但内容完全相同的版本。
2. **保持原文措辞:** 完全保留所有的原文措辞、短语、医学术语，甚至是拼写错误。不要进行任何修正或标准化处理。
3. **完整且静态的结构:** 你必须输出包含所有指定标题的完整 Markdown 模板。如果某个章节在原文中没有对应内容，你必须保留该标题，并将内容区域留空。
4. **无额外文本:** 你的输出必须只包含 Markdown 文档本身。不要在 Markdown 内容前后添加任何解释、笔记或对话性文字。

请根据你的系统指令处理下方提供的医疗记录。请精确地遵循示例所演示的格式。

---
**示例 (EXAMPLE)**

<示例原始记录>
入院诊断：1. 冠心病。出院医嘱：1. 按时服药。2. 定期复查。
</示例原始记录>

<示例预期输出>
# 病历记录

## 【入院时病情摘要】

## 【入院诊断】
1. 冠心病。

## 【住院诊疗经过】

## 【出院诊断】

## 【出院时情况】

## 【出院医嘱】
1. 按时服药。
2. 定期复查。

## 【补充说明】
</示例预期输出>
```

任务 (TASK)

现在，请处理以下新的记录：

<原始记录文本>

{raw_text}

</原始记录文本>

```
```

2.3. Prompt for Thermosis Event Extraction (English Version)

```
# SYSTEM ROLE DEFINITION
SYSTEM_ROLE = """You are an experienced medical expert specializing in the precise identification of thrombotic and bleeding events from clinical records. You must adhere to the following rules:
1. Strictly differentiate between events occurring during the current admission and past medical history. Only analyze and extract new or active events from the current course of treatment.
2. All your judgments must be based directly on the provided medical text, and you must cite key original text as evidence. Any form of speculation or use of external knowledge is prohibited.
3. Strictly follow the provided diagnostic criteria for thrombotic events (e.g., ISTH guidelines) for judgment and classification.
4. Output the results in JSON format, ensuring all fields are accurately populated."""

# PROMPT FOR THROMBOTIC EVENT EXTRACTION
THROMBOSIS_PROMPT = f"""
## Rules for Thrombosis Identification
[Inclusion Criteria]
- Confirmed by imaging: Reports from ultrasound, CT, MRI, angiography, etc., explicitly mentioning "thrombus," "embolism," "filling defect," or similar key descriptions.
- Confirmed by pathology or clinical diagnosis: A definitive diagnosis provided by a physician in progress notes, discharge summaries, or death records.
- Strong supportive evidence combination: When direct imaging confirmation is absent, the simultaneous presence of all three of the following: "initiation of therapeutic-dose anticoagulation/thrombolysis (e.g., heparin, warfarin, rivaroxaban)," "typical clinical symptoms," and "significantly abnormal laboratory markers (e.g., a sharp increase in D-dimer)."

[Exclusion Criteria]
- Past medical history: Thrombi explicitly described as "previous," "old," "chronic," "resolved," or occurring before the current admission.
- Risk assessment only: Mentions of risk alone, such as "Elevated D-dimer, be alert for thrombosis" or "High Wells score."
- Uncertain descriptions: Use of ambiguous terms like "suspected," "possible," "cannot be ruled out" without subsequent confirmatory tests or treatment. These should be marked as "Suspected" for further review.
- Procedure-related: For instance, "catheter-related thrombus" without clinical significance, or for prophylactic purposes.

## Diagnostic Criteria for Thrombotic Events (ISTH Guidelines)
### 1. Venous Thromboembolism (VTE) Diagnostic Criteria
- **Deep Vein Thrombosis (DVT)**:
  - **Diagnostic Standard**: Ultrasound/CT/MRI showing an intravascular filling defect or interruption of blood flow.
  - **Clinical Severity**:
    - **Mild**: Minor local symptoms, not affecting daily activities.
    - **Moderate**: Obvious symptoms, limiting daily activities.
    - **Severe**: Severe symptoms, completely limiting self-care or associated with a threat of limb ischemia.
  - **Risk Assessment**: Wells score ≥2 is considered "DVT Likely."

- **Pulmonary Embolism (PE)**:
```

  - **Diagnostic Standard**: CT Pulmonary Angiography (CTPA) showing a filling defect.
  - **Clinical Severity**:
    - **Low-risk**: No hemodynamic instability, PESI class I-II.
    - **Intermediate-risk**: Right ventricular dysfunction but normal blood pressure.
    - **High-risk**: Associated with shock or persistent hypotension.
  - **Risk Assessment**: Revised Geneva score or Wells score.

2. Arterial Thrombotic Event Diagnostic Criteria
- **Acute Coronary Syndrome (ACS)**:
  - **Diagnostic Standard**: Elevated cardiac markers + ECG changes / clinical symptoms.
  - **Classification**: STEMI, NSTEMI, Unstable Angina.

- **Ischemic Stroke**:
  - **Diagnostic Standard**: Head CT/MRI showing an ischemic lesion + neurological symptoms.
  - **Classification**: TOAST classification system (Large-artery atherosclerosis, Cardioembolism, Small-vessel occlusion, etc.).

- **Peripheral Artery Thrombosis**:
  - **Diagnostic Standard**: Angiography/CTA/MRA showing vessel occlusion.
  - **Classification**: Rutherford classification (Class 0-6).

3. Thrombosis in Special Sites
- **Portal Vein System Thrombosis**:
  - **Diagnostic Standard**: Ultrasound/CT/MRI showing a filling defect in the portal venous system.
  - **Classification**: Yerdel classification (Grade I-IV).

- **Cerebral Venous Sinus Thrombosis (CVST)**:
  - **Diagnostic Standard**: MRV/CTV showing a filling defect in a venous sinus.
  - **Classification**: Based on the number of affected sinuses and clinical presentation.

Scoring and Classification Standards Explained
Wells Score (for DVT)
- **Purpose**: To assess the clinical probability of suspected Deep Vein Thrombosis (DVT).
- **Criteria**:
    - Active cancer (treatment ongoing or within 6 months, or palliative): **+1 point**
    - Paralysis, paresis, or recent plaster immobilization of the lower extremities: **+1 point**
    - Recently bedridden for ≥3 days or major surgery within 12 weeks: **+1 point**
    - Localized tenderness along the distribution of the deep venous system: **+1 point**
    - Entire leg swollen: **+1 point**
    - Calf swelling >3 cm compared to the asymptomatic leg (measured 10 cm below tibial tuberosity): **+1 point**
    - Pitting edema confined to the symptomatic leg: **+1 point**
    - Collateral superficial veins (non-varicose): **+1 point**
    - An alternative diagnosis is at least as likely as DVT: **-2 points**
- **Interpretation (Two-level model)**:

    - **≥2 points**: **DVT Likely**
    - **<2 points**: **DVT Unlikely**

Wells Score (for PE)
- **Purpose**: To assess the clinical probability of suspected Pulmonary Embolism (PE).
- **Criteria**:
    - Clinical signs and symptoms of DVT: **+3 points**
    - PE is the #1 diagnosis OR equally likely: **+3 points**
    - Heart rate > 100 bpm: **+1.5 points**
    - Immobilization at least 3 days OR surgery in the previous 4 weeks: **+1.5 points**
    - Previous, objectively diagnosed DVT or PE: **+1.5 points**
    - Hemoptysis: **+1 point**
    - Malignancy with treatment within 6 months or palliative: **+1 point**
- **Interpretation (Two-level model)**:
    - **>4 points**: **PE Likely**
    - **≤4 points**: **PE Unlikely**

PESI / sPESI Score (Pulmonary Embolism Severity Index)
- **Purpose**: Prognostic assessment for **confirmed** PE patients (30-day mortality risk), **not for diagnosis**.
- **sPESI (Simplified version)**:
    - Age > 80 years: **+1 point**
    - History of cancer: **+1 point**
    - History of chronic cardiopulmonary disease: **+1 point**
    - Heart rate ≥ 110 bpm: **+1 point**
    - Systolic blood pressure < 100 mmHg: **+1 point**
    - Arterial oxygen saturation < 90%: **+1 point**
- **sPESI Interpretation**:
    - **0 points**: **Low Risk**
    - **≥1 point**: **High Risk**

TOAST Classification (for Ischemic Stroke Etiology)
- **Purpose**: To classify the etiology of a **confirmed** ischemic stroke.
- **Classification**:
    - **Large-artery atherosclerosis**: Imaging confirms ≥50% stenosis or occlusion of a relevant intracranial or extracranial artery, with no evidence of cardioembolism.
    - **Cardioembolism**: Presence of a high-risk cardiac source of embolism (e.g., atrial fibrillation, intracardiac thrombus, recent MI, valvular disease) with no evidence of large-artery atherosclerosis.
    - **Small-vessel occlusion (lacunar)**: Presence of a typical clinical lacunar syndrome, with an infarct <1.5 cm in diameter on imaging in a corresponding location, and no evidence of other causes.
    - **Stroke of other determined etiology**: Refers to rare causes like arterial dissection, vasculitis, hypercoagulable states, etc.
    - **Stroke of undetermined etiology**: The cause remains unclear after a comprehensive evaluation, or more than one potential cause is identified.

Rutherford Classification (for Acute Limb Ischemia)
- **Purpose**: To assess the severity of acute peripheral arterial ischemia.
- **Classification**:
    - **Class I (Viable)**: Not immediately threatened. No sensory loss, no muscle weakness. Arterial and venous Doppler signals are audible.
    - **Class IIa (Marginally Threatened)**: Salvageable if promptly treated. Minimal sensory loss (toes only), no muscle weakness. Arterial Doppler is often inaudible, venous is audible.
    - **Class IIb (Immediately Threatened)**: Salvageable with immediate intervention. Sensory loss more than toes, rest pain, mild to moderate muscle weakness. Arterial Doppler is inaudible, venous is audible.
    - **Class III (Irreversible)**: Major tissue loss or permanent nerve damage inevitable. Profound sensory loss, paralysis, and rigor. Arterial and venous Doppler signals are absent.

Yerdel Classification (for Portal Vein System Thrombosis)
- **Purpose**: To assess the anatomical extent of portal vein system thrombosis.
- **Classification**:
    - **Grade I**: Thrombosis confined to the main portal vein.
    - **Grade II**: Thrombosis extends to the main portal vein and one of its first-order branches (e.g., superior mesenteric or splenic vein).
    - **Grade III**: Thrombosis involves the main portal vein and both first-order branches.
    - **Grade IV**: Extensive thrombosis involving the entire portal and superior mesenteric veins.

Output Requirements
{{
  "thrombosis_events": [{{
    "certainty": "Confirmed/Ruled Out/Suspected",
    "type": "Deep Vein Thrombosis/Pulmonary Embolism/Acute Coronary Syndrome/Ischemic Stroke/...",
    "severity": "The specific classification or severity of the event (e.g., 'Low Risk', 'STEMI', 'Rutherford Class III') or 'Not specified'",
    "evidence": ["Direct quotes from the original text supporting the diagnosis (e.g., imaging report conclusions, diagnostic records, orders for therapeutic-dose medication and the reason)."],
    "risk_factors": ["Risk factors explicitly mentioned in the medical history or assessment related to this event (e.g., 'nephrotic syndrome', 'antiphospholipid syndrome', 'recent surgery')."]
  }}]
}}"""

# CONFLICT RESOLUTION RULES
CONFLICT_RULE = """
When conflicts arise:
1. Conflicting information between different records → Prioritize: Progress Notes > Nursing Records > Lab Reports.
2. Ambiguous time descriptions → Label as 'Time Unclear'.
3. Inconsistent terminology → Standardize according to ICD-11.
"""

TERMINOLOGY MAPPING

```
TERM_MAPPING = {
    "Thrombosis-related": {
        # Mapping common expressions to standard terms
        "Leg swelling": "Deep Vein Thrombosis",
        "Pulmonary infarction": "Pulmonary Embolism",
        "PE": "Pulmonary Embolism",
        "DVT": "Deep Vein Thrombosis",
        "VTE": "Venous Thromboembolism",
        "Cerebral infarction": "Ischemic Stroke",
        "Cerebral embolism": "Ischemic Stroke",
        "Heart attack": "Myocardial Infarction",
        "MI": "Myocardial Infarction",
        "AMI": "Acute Myocardial Infarction",
        "STEMI": "ST-Elevation Myocardial Infarction",
        "NSTEMI": "Non-ST-Elevation Myocardial Infarction",
        "Portal vein thrombus": "Portal Vein Thrombosis",
        "Mesenteric thrombus": "Mesenteric Vein Thrombosis",
        "Renal vein thrombus": "Renal Vein Thrombosis",
        "DIC": "Disseminated Intravascular Coagulation",
        "TMA": "Thrombotic Microangiopathy",
        "TTP": "Thrombotic Thrombocytopenic Purpura",
        "HIT": "Heparin-Induced Thrombocytopenia",
        "CAPS": "Catastrophic Antiphospholipid Syndrome",
        "Lower limb pain and swelling": "Suspected Deep Vein Thrombosis",
        "Limb coldness": "Suspected Arterial Thrombosis",
        "Sudden chest pain": "Suspected Pulmonary Embolism",
        "Sudden dyspnea": "Suspected Pulmonary Embolism",
        "Sudden loss of consciousness": "Suspected Ischemic Stroke",
        "Sudden limb weakness": "Suspected Ischemic Stroke",
        "Abdominal pain with diarrhea": "Suspected Mesenteric Thrombosis"
    }
}
```

FINAL OUTPUT INSTRUCTIONS
The output should be in JSON format only, without any markdown formatting.

You must provide the "thrombosis_events" output, even if no thrombotic events are found. If no events are found, output the following:

```
{
    "thrombosis_events": [{
        "certainty": "Ruled Out",
        "type": "None",
        "severity": "None",
        "evidence": ["No evidence of a thrombotic event was mentioned in the medical record."],
```

"risk_factors": []

}]

}

```
```

2.4. Prompt for Thrombosis Event Extraction (Chinese Version)

```
# 系统角色定义
SYSTEM_ROLE = """你是一位经验丰富的医学诊疗专家，负责精准识别临床病历中的出血与血栓事件。你需要：
1. 严格区分当次住院事件与既往病史，仅分析和提取本次诊疗过程中的新发或活动性事件
2. 你的所有判断必须直接基于提供的病历文本，并引用关键原文作为证据。禁止任何形式的推测或外部知识。
3. 严格按照下面提供的血栓事件诊断标准 (ISTH 指南)进行判断和分级。
4. 以 JSON 格式输出结果，确保所有字段都被准确填充。"""

# 血栓事件提取提示词
THROMBOSIS_PROMPT = f"""
## 血栓识别规则
【纳入标准】
- 影像学确诊：超声、CT、MRI、血管造影等影像学检查报告中明确提及“血栓”、“栓塞”、“充盈缺损”等关键描述。
- 病理或临床确诊：病程记录、出院小结或死亡记录中，由医生明确给出的诊断。
- 强支持性证据组合：当没有直接影像学确诊时，“启用治疗剂量的抗凝/溶栓药物 (如肝素、华法林、利伐沙班等) ” + “典型的临床症状” + “显著异常的实验室指标（如 D-Dimer 急剧升高）”三者同时满足。

【排除标准】
- 既往史：明确描述为“既往”、“陈旧性”、“已愈”或发生在本次入院前的血栓。
- 风险评估：仅提及风险，如“D-Dimer 升高，请警惕血栓形成”、“Wells 评分高”等。
- 不确定性描述：使用“可疑”、“可能”、“不除外”等模糊词语，且无后续检查或治疗证实的，这类应被标记为“疑似”以待后续查验。
- 操作相关：如“导管相关血栓”但无临床意义，或为预防性措施。

## 血栓事件诊断标准（ISTH 指南）
### 1. 静脉血栓栓塞症(VTE)诊断标准
- **深静脉血栓(DVT)**:
  - **确诊标准**：超声/CT/MRI 显示血管内充盈缺损或血流中断
  - **临床分级**:
    - **轻度**：局部症状轻微，不影响日常活动
    - **中度**：明显症状，限制日常活动
    - **重度**：严重症状，完全限制自理能力或伴有肢体缺血威胁
  - **风险评估**: Wells 评分≥2 分为"DVT 可能"

- **肺栓塞(PE)**:
  - **确诊标准**: CT 肺动脉造影(CTPA)显示充盈缺损
  - **临床分级**:
    - **低危**：无血流动力学不稳定，PESI 评分 I-II 级
    - **中危**：右心功能受损但血压正常
    - **高危**：伴有休克或持续性低血压
  - **风险评估**：修订版 Geneva 评分或 Wells 评分
```

2. 动脉血栓事件诊断标准
- **急性冠脉综合征**:
  - **确诊标准**: 心肌标志物升高+心电图改变/临床症状
  - **分类**: STEMI、NSTEMI、不稳定型心绞痛

- **缺血性脑卒中**:
  - **确诊标准**: 头颅 CT/MRI 显示缺血性病变+神经系统症状
  - **分类**: TOAST 分类系统(大动脉粥样硬化型、心源性栓塞型、小血管闭塞型等)

- **外周动脉血栓**:
  - **确诊标准**: 血管造影/CTA/MRA 显示血管闭塞
  - **分级**: Rutherford 分级(0-6 级)

3. 特殊部位血栓
- **门静脉系统血栓**:
  - **确诊标准**: 超声/CT/MRI 显示门静脉系统充盈缺损
  - **分级**: Yerdel 分级(I-IV 级)

- **脑静脉窦血栓**:
  - **确诊标准**: MRV/CTV 显示静脉窦充盈缺损
  - **分级**: 根据受累静脉窦数量和临床表现

评分标准解析
Wells 评分标准 (用于 DVT)
- **用途**: 评估疑似深静脉血栓（DVT）的临床可能性。
- **评分项**:
    - 活动性癌症 (正在接受治疗或 6 个月内接受过姑息治疗): **+1 分**
    - 下肢瘫痪、麻痹或近期石膏固定: **+1 分**
    - 近期卧床≥3 天或 4 周内接受过大手术: **+1 分**
    - 沿深静脉走行的局部压痛: **+1 分**
    - 整个下肢水肿: **+1 分**
    - 患侧小腿周径比健侧>3cm (在胫骨粗隆下 10cm 测量): **+1 分**
    - 患侧凹陷性水肿 (比健侧明显): **+1 分**
    - 非静脉曲张性浅表侧支静脉: **+1 分**
    - 存在比 DVT 可能性更大的其他诊断: **-2 分**
- **结果解读 (两水平法)**:
    - **≥2 分**: **DVT 可能 (Likely)**
    - **<2 分**: **DVT 不太可能 (Unlikely)**

Wells 评分标准 (用于 PE)
- **用途**: 评估疑似肺栓塞（PE）的临床可能性。
- **评分项**:
    - 临床体征和症状提示 DVT (至少有下肢水肿和触痛): **+3 分**
    - 除了 PE 没有其他更可能的诊断: **+3 分**

- 心率 ＞100 次/分: **+1.5 分**
- 过去 4 周内有制动或外科手术史: **+1.5 分**
- 既往有 DVT 或 PE 病史: **+1.5 分**
- 咯血: **+1 分**
- 恶性肿瘤 (正在治疗、姑息治疗或 6 个月内诊断): **+1 分**
- **结果解读 (两水平法)**:
- **>4 分**: **PE 可能 (Likely)**
- **≤4 分**: **PE 不太可能 (Unlikely)**

PESI / sPESI 评分标准 (肺栓塞严重程度指数)
- **用途**: **已确诊**PE 患者的预后评估（30 天死亡风险），**不用于诊断**。
- **sPESI (简化版)**:
- 年龄 ＞80 岁: **+1 分**
- 有癌症病史: **+1 分**
- 有慢性心肺疾病史: **+1 分**
- 心率 ≥110 次/分: **+1 分**
- 收缩压 ＜100 mmHg: **+1 分**
- 动脉血氧饱和度 ＜90%: **+1 分**
- **sPESI 结果解读**:
- **0 分**: **低危**
- **≥1 分**: **高危**

TOAST 分型标准 (缺血性脑卒中病因)
- **用途**: 对**已确诊**的缺血性脑卒中进行病因学分类。
- **分型标准**:
- **大动脉粥样硬化型**: 影像学证实相应供血区域的颅内或颅外大动脉存在≥50%的狭窄或闭塞，且无心源性栓塞证据。
- **心源性栓塞型**: 存在高风险的心源性栓子来源（如房颤、心内血栓、近期心梗、瓣膜病等），且无大动脉粥样硬化证据。
- **小血管闭塞型 (腔隙性)**: 存在典型的临床腔隙综合征，影像学上可见相应部位直径<1.5cm 的梗死灶，且无其他病因证据。
- **其他明确病因型**: 指少见原因，如动脉夹层、血管炎、高凝状态等。
- **不明原因型**: 经过全面评估后仍无法确定病因，或存在两种及以上可能病因。

Rutherford 分级标准 (急性肢体缺血)
- **用途**: 评估急性外周动脉缺血的严重程度。
- **分级标准**:
- **I 级 (Viable, 存活)**: 肢体未受威胁，无感觉丧失，无肌无力。动脉和静脉多普勒信号均可闻及。
- **IIa 级 (Marginally Threatened, 轻度受威胁)**: 可挽救，感觉轻度丧失（仅限于足趾），无肌无力。动脉多普勒信号常听不清，静脉信号可闻及。
- **IIb 级 (Immediately Threatened, 立即受威胁)**: 可挽救但需立即干预，感觉丧失超过足趾范围，静息痛，轻至中度肌无力。动脉多普勒信号听不清，静脉信号可闻及。
- **III 级 (Irreversible, 不可逆)**: 需截肢，肢体有麻痹和僵硬，感觉完全丧失。动脉和静脉多普勒信号均消失。

Yerdel 分级标准 (门静脉系统血栓)
- **用途**: 评估门静脉系统血栓的解剖范围。
- **分级标准**:
  - **I 级**: 血栓仅限于肝门静脉主干。
  - **II 级**: 血栓累及肝门静脉主干及其一级分支（如肠系膜上静脉或脾静脉)。
  - **III 级**: 血栓累及肝门静脉主干及其一、二级分支。
  - **IV 级**: 血栓广泛累及整个门静脉和肠系膜上静脉。

输出要求
{{
  "thrombosis_events": [{
    "certainty": "是/否/疑似",
    "type": "深静脉血栓/肺栓塞/急性冠脉综合征/缺血性脑卒中/...", # 根据诊断选择最具体类型
    "severity": "具体事件的标准分级或严重度 (如'低危'/'STEMI'/'Rutherford 3 级'/...) 或 '未明确'",
    "evidence": ["直接支持诊断的关键原文引用 (如影像报告结论、诊断记录、启用治疗剂量的医嘱及原因)"],
    "risk_factors": ["在本次事件相关的病史或评估中明确提及的风险因素（如'肾病综合征'、'抗磷脂综合征'、'近期手术史'等）"]
  }]
}}"""

# 矛盾处理规则
CONFLICT_RULE = """
当出现以下矛盾时:
1. 不同记录间信息冲突 → 按病程记录>护理记录>检验报告
2. 时间描述模糊 → 标注为"时间不明"
3. 术语不一致 → 按 ICD-11 标准统一"""

# 术语映射表
TERM_MAPPING = {
  "血栓相关": {
    # 常见表述映射到标准术语
    "腿肿": "下肢深静脉血栓",
    "肺梗": "肺栓塞",
    "PE": "肺栓塞",
    "DVT": "深静脉血栓",
    "VTE": "静脉血栓栓塞症",
    "脑梗": "脑动脉血栓",
    "脑栓塞": "脑动脉栓塞",
    "心梗": "心肌梗死",
    "AMI": "急性心肌梗死",
    "STEMI": "ST 段抬高型心肌梗死",
    "NSTEMI": "非 ST 段抬高型心肌梗死",
    "门静脉血栓": "门静脉血栓形成",
    "肠系膜血栓": "肠系膜静脉血栓形成",

    "肾静脉血栓": "肾静脉血栓形成",
    "DIC": "弥散性血管内凝血",
    "TMA": "血栓性微血管病",
    "TTP": "血栓性血小板减少性紫癜",
    "HIT": "肝素诱导的血小板减少症",
    "CAPS": "灾难性抗磷脂综合征",
    "下肢胀痛": "疑似下肢深静脉血栓",
    "肢体发凉": "疑似动脉血栓",
    "突发胸痛": "疑似肺栓塞",
    "突发呼吸困难": "疑似肺栓塞",
    "突发意识障碍": "疑似脑栓塞",
    "突发肢体无力": "疑似脑栓塞",
    "腹痛伴腹泻": "疑似肠系膜血栓"
  }
}

输出要求
输出仅为 jason 格式，不要纳入 markdown 格式内容

请务必提供 thrombosis_events 的输出，即使未发现出血事件。若无血栓事件，请输出：
{
  "thrombosis_events": [{
    "certainty": "否",
    "type": "无",
    "severity": "无",
    "evidence": ["病历中未提及任何血栓事件证据"],
    "risk_factors": []
  }]
}
```
```

2.5. Prompt for Bleeding Event Extraction (English Version)

```
# SYSTEM ROLE DEFINITION
SYSTEM_ROLE = """You are an experienced medical expert specializing in the precise identification of bleeding and thrombotic events from clinical records. You must adhere to the following rules:
1. Strictly differentiate between events occurring during the current admission and past medical history. Only analyze and extract new or active events from the current course of treatment.
2. All your judgments must be based directly on the provided medical text, and you must cite key original text as evidence. Any form of speculation or use of external knowledge is prohibited.
3. Strictly follow the provided diagnostic criteria for bleeding events (e.g., CTCAE, ISTH guidelines) for judgment and classification.
4. Output the results in JSON format, ensuring all fields are accurately populated."""

# PROMPT FOR BLEEDING EVENT EXTRACTION
BLEEDING_PROMPT = f"""
## Rules for Bleeding Identification
[Inclusion Criteria]
- **Objective Evidence**: Reports from CT, MRI, angiography, etc., explicitly mentioning **active contrast extravasation** or a **new/enlarging hematoma**; direct observation during endoscopy (e.g., gastroscope, bronchoscope) of **active bleeding** (e.g., spurting, oozing) or **stigmata of recent hemorrhage** (e.g., non-bleeding visible vessel, adherent clot).
- **Clinical Intervention**: Transfusion of **packed red blood cells (PRBCs) or whole blood** to correct blood loss; invasive procedures such as **surgery or interventional embolization** to achieve hemostasis; presence of **persistent hypotension (systolic BP < 90 mmHg) or tachycardia (heart rate > 110 bpm)** directly related to bleeding and requiring **vasoactive agents or fluid resuscitation**.
- **Critical Site Bleeding**: Any confirmed bleeding in a **critical site** (intracranial, intraspinal, intrapericardial, retroperitoneal, intra-articular), regardless of volume; presence of clear macroscopic bleeding symptoms such as **hematemesis, melena, hemoptysis, or gross hematuria** (can be initially marked as "Suspected" pending further confirmation).

[Exclusion Criteria]
- **Expected/Controlled Procedural Bleeding**: Minor oozing from surgical incisions, small amounts of bleeding after drain removal, or ecchymosis at routine puncture sites. However, **unexpected procedural bleeding that requires additional intervention (e.g., reoperation for hemostasis, blood transfusion) should be included**.
- **Physiological Bleeding**: Such as normal menstruation in female patients.
- **Clinically Insignificant Lab Abnormalities**: Isolated positive **fecal or urine occult blood tests** without any other clinical symptoms, signs, or a drop in hemoglobin.
- **Minor Mucocutaneous Manifestations**: Scattered **petechiae or ecchymoses** less than 5 cm in diameter with no tendency to expand; minor gingival oozing or blood-streaked nasal discharge.

## Diagnostic Criteria for Bleeding Events (CTCAE/ISTH Guidelines)
### 1. CTCAE v5.0 Grading for Hemorrhage
```

- **Grade 1 (Mild)**: Mild symptoms; observation only; intervention not indicated.
- **Grade 2 (Moderate)**: Local intervention indicated; limiting instrumental Activities of Daily Living (ADL); transfusion may be needed.
- **Grade 3 (Severe)**: Transfusion, endoscopic, or surgical intervention indicated; limiting self-care ADL; hemodynamic instability.
- **Grade 4 (Life-threatening)**: Urgent intervention indicated; requires intensive care; multi-organ dysfunction.
- **Grade 5 (Death)**: Death.

2. ISTH Bleeding Severity Classification
- **Minor Bleeding**: Does not meet the criteria for major or clinically relevant non-major bleeding (CRNMB).
- **Clinically Relevant Non-Major Bleeding (CRNMB)**: Does not meet major bleeding criteria but requires medical intervention, leads to hospitalization, requires a face-to-face evaluation, or causes temporary cessation of activities.
- **Major Bleeding**: Fatal bleeding; symptomatic bleeding in a critical area or organ (intracranial, intraspinal, intraocular, pericardial, intra-articular, retroperitoneal, or intramuscular with compartment syndrome); causes a fall in hemoglobin of ≥2 g/dL (20 g/L) or requires transfusion of ≥2 units of packed red cells.

3. Site-Specific Bleeding Criteria
- **Intracranial Hemorrhage**: Bleeding focus shown on head CT/MRI; assess with ICH Score (0-6).
- **Gastrointestinal (GI) Bleeding**: Bleeding source confirmed by endoscopy or clear clinical evidence; assess with Forrest classification (upper GI) or BLEED score.
- **Urogenital Bleeding**: Gross hematuria, positive dipstick test + microscopic RBCs, imaging/endoscopic evidence; graded by hemoglobin drop and need for intervention.
- **Respiratory Bleeding (Hemoptysis)**: Clinical symptoms + source confirmed by bronchoscopy/CT. **Mild**: <5mL/24h, blood-streaked sputum; **Moderate**: 5-100mL/24h, no respiratory compromise; **Severe**: 100-600mL/24h, potential respiratory compromise; **Massive**: >600mL/24h or >100mL/h, life-threatening.

Scoring and Classification Standards Explained
ICH Score (for Intracerebral Hemorrhage)
- **Purpose**: To assess the severity and predict 30-day mortality for patients with intracerebral hemorrhage (ICH).
- **Criteria**:
  - **Glasgow Coma Scale (GCS)**: 3-4 (**+2 points**), 5-12 (**+1 point**), 13-15 (**+0 points**)
  - **ICH Volume**: ≥30 cm^3 (**+1 point**), <30 cm^3 (**+0 points**)
  - **Intraventricular Hemorrhage (IVH)**: Present (**+1 point**), Absent (**+0 points**)
  - **Infratentorial Origin (brainstem or cerebellum)**: Present (**+1 point**), Absent (**+0 points**)
  - **Age**: ≥80 years (**+1 point**), <80 years (**+0 points**)
- **Interpretation**: Total score 0-6; higher scores indicate worse prognosis.

Forrest Classification (for Upper GI Ulcer Bleeding)
- **Purpose**: To assess the risk of re-bleeding from peptic ulcers based on endoscopic findings.
- **Classification**:
  - **Active Bleeding**:
    - **Ia**: Spurting arterial hemorrhage
    - **Ib**: Oozing hemorrhage
  - **Stigmata of Recent Hemorrhage**:
    - **IIa**: Non-bleeding visible vessel
    - **IIb**: Adherent clot
    - **IIc**: Flat pigmented spot (hematin) on ulcer base
  - **No Active Bleeding**:
    - **III**: Clean ulcer base

BLEED Score
- **Purpose**: To assess the risk of re-bleeding and mortality in patients with acute non-variceal upper GI bleeding.
- **Criteria**:
  - **B - Ongoing Bleeding**: Bright red hematemesis or hematochezia, or hemodynamic instability: **+1 point**
  - **L - Low systolic blood pressure**: SBP < 100 mmHg at presentation: **+1 point**
  - **E - Elevated prothrombin time**: >1.2 times normal: **+1 point**
  - **E - Erratic mental status or unstable comorbid disease**: **+1 point**
  - **D - Age > 60 years**: **+1 point**
- **Interpretation**: Higher scores indicate higher risk; a score ≥3 is typically considered high risk.

Output Requirements
{{
  "bleeding_events": [{{
    "certainty": "Confirmed/Ruled Out/Suspected",
    "type": "Gastrointestinal/Intracranial/Urogenital/etc.",
    "severity": "Mild/Moderate/Severe/Life-threatening/Death/Unknown",
    "evidence": ["Quote from original text"],
    "risk_factors": ["Thrombocytopenia (<50x10^9/L), etc."]
  }}]
}}"""

# CONFLICT RESOLUTION RULES
CONFLICT_RULE = """
When conflicts arise:
1. Conflicting information between different records → Prioritize: Progress Notes > Nursing Records > Lab Reports.
2. Ambiguous time descriptions → Label as 'Time Unclear'.
3. Inconsistent terminology → Standardize according to ICD-11.

```
"""

# TERMINOLOGY MAPPING
TERM_MAPPING = {
    "Bleeding-related": {
        # Mapping common expressions to standard terms
        "Melena": "Gastrointestinal Bleeding",
        "Hematemesis": "Upper Gastrointestinal Bleeding",
        "Hematochezia": "Lower Gastrointestinal Bleeding",
        "Hemoptysis": "Respiratory Bleeding",
        "Hematuria": "Urogenital Bleeding",
        "Epistaxis": "Nasal Bleeding",
        "Gingival bleeding": "Oral Bleeding",
        "Ecchymosis": "Mucocutaneous Bleeding",
        "Petechiae": "Mucocutaneous Bleeding",
        "Purpura": "Mucocutaneous Bleeding",
        "Cerebral hemorrhage": "Intracranial Hemorrhage",
        "SAH": "Subarachnoid Hemorrhage",
        "SDH": "Subdural Hematoma",
        "EDH": "Epidural Hematoma",
        "ICH": "Intracerebral Hemorrhage",
        "IVH": "Intraventricular Hemorrhage",
        "Hemothorax": "Thoracic Bleeding",
        "Hemopneumothorax": "Thoracic Bleeding",
        "Hemorrhagic pleural effusion": "Pleural Effusion with Bleeding",
        "Hemorrhagic ascites": "Ascites with Bleeding",
        "Retroperitoneal hematoma": "Retroperitoneal Bleeding",
        "Hemarthrosis": "Intra-articular Bleeding",
        "Muscle hematoma": "Muscular Bleeding",
        "Vaginal bleeding": "Genital Bleeding",
        "Subconjunctival hemorrhage": "Ocular Bleeding",
        "Retinal hemorrhage": "Ocular Bleeding",
        "Vitreous hemorrhage": "Ocular Bleeding"
    }
}
```

FINAL OUTPUT INSTRUCTIONS
The output should be in JSON format only, without any markdown formatting.

You must provide the "bleeding_events" output, even if no bleeding events are found. If no events are found, output the following:

```
{
    "bleeding_events": [{
        "certainty": "Ruled Out",
```

"type": "None",
"severity": "None",
"evidence": ["No evidence of a bleeding event was mentioned in the medical record."],
"risk_factors": []
}]
}
```
```

2.6. Prompt for Bleeding Event Extraction (Chinese Version)

```
# 系统角色定义
SYSTEM_ROLE = """你是一位经验丰富的医学诊疗专家，负责精准识别临床病历中的出血与血栓事件。你需要：
1. 严格区分当次住院事件与既往病史，仅分析和提取本次诊疗过程中的新发或活动性事件
2. 你的所有判断必须直接基于提供的病历文本，并引用关键原文作为证据。禁止任何形式的推测或外部知识。
3. 严格按照下面提供的血栓事件诊断标准 (ISTH 指南)进行判断和分级。
4. 以 JSON 格式输出结果，确保所有字段都被准确填充。"""

# 出血事件提取提示词
BLEEDING_PROMPT = f"""
## 出血识别规则
【纳入标准】
- 客观证据：CT、MRI、血管造影等明确报告存在活动性造影剂外渗、新发或增大的血肿；胃肠镜、支气管镜等直视下观察到活动性出血（如喷射性、渗出性）或近期出血征象（如裸露血管、附着血凝块）。
- 临床干预：输注了红细胞悬液或全血以纠正失血；采取了如手术或介入栓塞等有创方式进行止血；出现与出血直接相关的持续性低血压（收缩压<90mmHg）或心动过速（心率>110 次/分），且需要血管活性药物或液体复苏支持。
- 重要脏器出血：任何明确的颅内、椎管内、心包、腹膜后、关节腔内的出血，无论出血量大小；出现如呕血、黑便、咯血、肉眼血尿等明确的宏观出血症状（可先标记为“疑似”，待进一步证据证实）。

【排除标准】
- 可预期的、在控制范围内的操作性出血：如手术切口的少量渗血、拔除引流管后的少量出血、常规穿刺点的皮下瘀斑等。但超出预期的、导致额外干预（如二次手术止血、输血）的操作相关出血应被纳入。
- 生理性出血：如女性患者的正常月经。
- 无临床意义的实验室异常：单纯的大便或尿液潜血阳性，而无任何其他临床症状、体征或血红蛋白下降。
- 轻微的皮肤黏膜表现：直径小于 5cm 且无扩展趋势的散在皮下瘀点、瘀斑；轻微的牙龈渗血或鼻涕带血。

## 出血事件诊断标准（CTCAE/ISTH 指南）
### 1. CTCAE 出血事件分级(v5.0)
- **1 级(轻度)**：轻微症状，仅临床观察，不需要干预
- **2 级(中度)**：需要局部干预，限制日常生活活动，可能需要输血
- **3 级(重度)**：需要输血/内镜/手术干预，限制自理能力，血流动力学不稳定
- **4 级(危及生命)**：需要紧急手术干预，需要重症监护，多器官功能障碍
- **5 级**：死亡

### 2. ISTH 出血严重程度分级
- **轻微出血**：不符合重大或非重大临床相关出血标准
- **非重大临床相关出血(CRNMB)**：不符合重大出血标准但需要医疗干预，导致住院，需要面对面评估，导致活动暂时中断
- **重大出血(Major Bleeding)**：致命性出血，关键部位出血(颅内、脊髓内、眼内、心包、关节内、腹膜后、肌肉内伴有筋膜综合征)，导致血红蛋白下降≥20g/L 或需要输注≥2U 红细胞

### 3. 特定部位出血标准
```

- **颅内出血**: 头颅 CT/MRI 显示出血灶，ICH 评分(0-6 分)
- **消化道出血**: 内镜确认出血源或临床明确证据，Forrest 分级(上消化道)或 BLEED 评分
- **泌尿系统出血**: 肉眼血尿，干化学法隐血阳性+镜检见红细胞，影像学/内镜证据，根据血红蛋白下降程度和临床干预需求分级
- **呼吸系统出血(咯血)**: 临床症状+支气管镜/CT 确认出血源。**轻度**: <5mL/24h，少量血丝痰;**中度**: 5-100mL/24h，不影响呼吸功能;**重度**: 100-600mL/24h，可能影响呼吸功能;**大量**: >600mL/24h 或>100mL/h，威胁生命

评分/分级标准解析
ICH 评分 (脑实质出血)
- **用途**: 评估脑实质出血（ICH）患者的严重程度及预测 30 天死亡率。
- **评分项**:
    - **格拉斯哥昏迷评分 (GCS)**: 3-4 分(**+2 分**), 5-12 分(**+1 分**), 13-15 分(**+0 分**)
    - **出血体积**: ≥30 cm^3(**+1 分**), <30 cm^3(**+0 分**)
    - **脑室内出血**: 有(**+1 分**), 无(**+0 分**)
    - **幕下出血 (脑干或小脑)**: 有(**+1 分**), 无(**+0 分**)
    - **年龄**: ≥80 岁(**+1 分**), <80 岁(**+0 分**)
- **结果解读**: 总分 0-6 分，分数越高，预后越差。

Forrest 分级 (上消化道溃疡出血)
- **用途**: 通过内镜下表现评估消化性溃疡的再出血风险。
- **分级标准**:
    - **活动性出血**:
        - **Ia 级**: 动脉喷射性出血
        - **Ib 级**: 渗出性出血
    - **近期出血痕迹**:
        - **IIa 级**: 可见裸露血管
        - **IIb 级**: 附着有血凝块
        - **IIc 级**: 溃疡基底有血红素或黑点
    - **无活动性出血**:
        - **III 级**: 溃疡基底干净

BLEED 评分
- **用途**: 评估急性非静脉曲张性上消化道出血患者的再出血风险和死亡风险。
- **评分项**:
    - **B - 持续性出血**: 鲜红色呕血或便血，或生命体征不稳定: **+1 分**
    - **L - 低收缩压**: 就诊时收缩压<100mmHg: **+1 分**
    - **E - 凝血酶原时间延长**: >1.2 倍正常值: **+1 分**
    - **E - 不稳定的合并症**: 存在精神状态改变或不稳定的基础疾病: **+1 分**
    - **D - 年龄 > 60 岁**: **+1 分**
- **结果解读**: 总分越高，风险越高，通常≥3 分被认为是高风险。

输出要求
{{

```
    "bleeding_events": [{
        "certainty": "是/否/疑似",
        "type": "消化道/颅内/泌尿系统等",
        "severity": "轻度/中度/重度/危及生命/死亡/未知",
        "evidence": ["原文引用"],
        "risk_factors": ["血小板<50×10^9/L 等"]
    }]
}}"""

# 矛盾处理规则
CONFLICT_RULE = """
当出现以下矛盾时：
1. 不同记录间信息冲突 → 按病程记录>护理记录>检验报告
2. 时间描述模糊 → 标注为"时间不明"
3. 术语不一致 → 按 ICD-11 标准统一"""

# 术语映射表
TERM_MAPPING = {
    "出血相关": {
        # 常见表述映射到标准术语
        "黑便": "消化道出血",
        "呕血": "上消化道出血",
        "便血": "下消化道出血",
        "咯血": "呼吸道出血",
        "血尿": "泌尿系统出血",
        "鼻衄": "鼻出血",
        "牙龈出血": "口腔出血",
        "皮下瘀斑": "皮肤黏膜出血",
        "瘀点": "皮肤黏膜出血",
        "紫癜": "皮肤黏膜出血",
        "脑出血": "颅内出血",
        "SAH": "蛛网膜下腔出血",
        "SDH": "硬膜下出血",
        "EDH": "硬膜外出血",
        "ICH": "脑实质出血",
        "IVH": "脑室内出血",
        "血胸": "胸腔出血",
        "血气胸": "胸腔出血",
        "血性胸水": "胸腔积液伴出血",
        "血性腹水": "腹腔积液伴出血",
        "腹膜后血肿": "腹膜后出血",
        "关节血肿": "关节腔出血",
        "肌肉血肿": "肌肉出血",
        "阴道流血": "生殖系统出血",
```

"结膜下出血": "眼部出血",
"视网膜出血": "眼部出血",
"玻璃体出血": "眼部出血",
}
}

输出要求
输出仅为 jason 格式，不要纳入 markdown 格式内容

请务必提供 bleeding_events 的输出，即使未发现出血事件。若无出血事件，请输出：
{
"bleeding_events": [{
"certainty": "否",
"type": "无",
"severity": "无",
"evidence": ["病历中未提及任何出血事件证据"],
"risk_factors": []
}]
}
```
```

Supplementary Table S1. Complete Baseline Demographic and Clinical Characteristics of the Study Cohorts.

Characteristic	N (Riva, Indo, Clop)	Data Type	Unit	Rivaroxaban	Indobufen	Clopidogrel	Statistical Method	P-value	FDR P-value
Hypocalcemia at Admission	453 (72, 286, 95)	n (%)		72 (75.8%)	286 (79.9%)	95 (36.1%)	Chi-square test	< 0.001	< 0.001
Heavy Proteinuria at Admission	286 (54, 198, 34)	n (%)		54 (56.8%)	198 (55.3%)	34 (12.9%)	Chi-square test	< 0.001	< 0.001
Hypercoagulability at Admission	446 (71, 281, 94)	n (%)		71 (74.7%)	281 (78.5%)	94 (35.7%)	Chi-square test	< 0.001	< 0.001
Nephrotic Syndrome	546 (84, 312, 150)	n (%)		84 (88.4%)	312 (87.2%)	150 (57.0%)	Chi-square test	< 0.001	< 0.001
Coronary Heart Disease	106 (7, 31, 68)	n (%)		7 (7.4%)	31 (8.7%)	68 (25.9%)	Chi-square test	< 0.001	< 0.001
Age	716 (132, 411, 231)	Median (IQR)	years	47.00 (28.00, 66.50)	42.50 (19.00, 60.00)	57.00 (41.00, 66.00)	Kruskal-Wallis test	< 0.001	< 0.001
ALB	251 (42, 138, 71)	Median (IQR)	g/L	20.10 (17.18, 35.62)	19.75 (14.57, 25.17)	29.50 (24.05, 36.40)	Kruskal-Wallis test	< 0.001	< 0.001
Edema	558 (76, 308, 174)	n (%)		76 (80.0%)	308 (86.0%)	174 (66.2%)	Chi-square test	< 0.001	< 0.001

Characteristic	N (Riva, Indo, Clop)	Data Type	Unit	Rivaroxaban	Indobufen	Clopidogrel	Statistical Method	P-value	FDR P-value
Risk of Iron Overload at Admission	148 (27, 97, 24)	n (%)		27 (28.4%)	97 (27.1%)	24 (9.1%)	Chi-square test	< 0.001	< 0.001
Calcium	544 (86, 316, 142)	Median (IQR)	mmol/L	1.93 (1.85, 2.08)	1.96 (1.85, 2.06)	2.06 (1.92, 2.20)	Kruskal-Wallis test	< 0.001	< 0.001
Hyperlipidemia	602 (81, 325, 196)	n (%)		81 (85.3%)	325 (90.8%)	196 (74.5%)	Chi-square test	< 0.001	< 0.001
Red Blood Cell Count	526 (79, 307, 140)	Mean ± SD	10^{12}/L	4.63 ± 1.11	4.27 ± 1.25	3.86 ± 0.92	Welch-ANOVA	< 0.001	< 0.001
Total Protein	246 (42, 133, 71)	Median (IQR)	g/L	43.40 (37.58, 59.55)	42.80 (37.40, 51.30)	57.60 (48.65, 67.65)	Kruskal-Wallis test	< 0.001	< 0.001
PT	489 (73, 285, 131)	Median (IQR)	seconds	10.71 (10.10, 11.80)	11.60 (10.30, 12.70)	12.10 (11.00, 12.90)	Kruskal-Wallis test	< 0.001	< 0.001
Urine Dipstick Protein at Admission	416 (97, 249, 70)	[Negative, Trace, Positive, Strong Positive]		10(10.3%), 1(1.0%), 3(3.1%), 83(85.6%)	7(2.8%), 3(1.2%), 12(4.8%), 227(91.2%)	23(32.9%), 1(1.4%), 7(10.0%), 39(55.7%)	Kruskal-Wallis test (for ordered data)	< 0.001	< 0.001

Characteristic	N (Riva, Indo, Clop)	Data Type	Unit	Rivaroxaban	Indobufen	Clopidogrel	Statistical Method	P-value	FDR P-value
Urine Dipstick Occult Blood at Admission	446 (97, 252, 97)	[Negative, Trace, Positive, Strong Positive]		23(23.7%), 14(14.4%), 27(27.8%), 33(34.0%)	35(13.9%), 44(17.5%), 77(30.6%), 96(38.1%)	40(41.2%), 31(32.0%), 14(14.4%), 12(12.4%)	Kruskal-Wallis test (for ordered data)	< 0.001	< 0.001
Total Cholesterol at Admission	558 (110, 306, 142)	[Normal, Mildly elevated, Markedly elevated]		23(20.9%), 14(12.7%), 73(66.4%)	65(21.2%), 38(12.4%), 203(66.3%)	60(42.3%), 27(19.0%), 55(38.7%)	Kruskal-Wallis test (for ordered data)	< 0.001	< 0.001
Albumin/Globulin Ratio	245 (42, 133, 70)	Mean ± SD	ratio	1.07 ± 0.42	0.89 ± 0.37	1.16 ± 0.34	One-way ANOVA	< 0.001	< 0.001
MPV	514 (75, 301, 138)	Median (IQR)	fL	9.20 (8.55, 9.95)	9.20 (8.70, 9.80)	9.75 (9.12, 10.30)	Kruskal-Wallis test	< 0.001	< 0.001
Focal Segmental Glomerulosclerosis (FSGS)	75 (8, 57, 10)	n (%)		8 (8.4%)	57 (15.9%)	10 (3.8%)	Chi-square test	< 0.001	< 0.001
CK-MB	450 (66, 265, 119)	Median (IQR)	U/L	21.80 (16.18, 30.10)	23.20 (17.30, 36.10)	18.00 (14.65, 24.80)	Kruskal-Wallis test	< 0.001	< 0.001
Total Cholesterol	165 (28, 97, 40)	Median (IQR)	mmol/L	10.18 (6.65, 12.41)	9.00 (6.37, 12.12)	5.79 (4.84, 7.86)	Kruskal-Wallis test	< 0.001	0.001

Characteristic	N (Riva, Indo, Clop)	Data Type	Unit	Rivaroxaban	Indobufen	Clopidogrel	Statistical Method	P-value	FDR P-value
Leucine Aminopeptidase	240 (41, 130, 69)	Median (IQR)	U/L	41.00 (34.00, 51.70)	39.95 (32.12, 51.83)	32.00 (25.40, 37.40)	Kruskal-Wallis test	< 0.001	0.001
GLU	387 (67, 220, 100)	Median (IQR)	mmol/L	5.54 (4.40, 6.55)	5.21 (4.51, 6.32)	6.24 (5.08, 9.57)	Kruskal-Wallis test	< 0.001	0.001
Low_Density Lipoprotein Cholesterol	162 (26, 97, 39)	Median (IQR)	mmol/L	6.54 (4.04, 8.77)	6.32 (4.13, 8.69)	3.74 (2.88, 5.47)	Kruskal-Wallis test	< 0.001	0.001
Lower Limb Edema	539 (72, 297, 170)	n (%)		36 (37.9%)	136 (38.0%)	81 (30.8%)	Chi-square test	< 0.001	0.001
Uremia	55 (0, 40, 15)	n (%)		0 (0.0%)	40 (11.2%)	15 (5.7%)	Fisher's exact test (with simulated p-value)	< 0.001	0.001
CKD Present at Admission	455 (52, 275, 128)	n (%)		11 (11.6%)	63 (17.6%)	23 (8.7%)	Fisher's exact test (with simulated p-value)	< 0.001	0.001
Nephrolithiasis	133 (14, 49, 70)	n (%)		14 (14.7%)	49 (13.7%)	70 (26.6%)	Chi-square test	< 0.001	0.001

Characteristic	N (Riva, Indo, Clop)	Data Type	Unit	Rivaroxaban	Indobufen	Clopidogrel	Statistical Method	P-value	FDR P-value
Pitting Edema	426 (60, 236, 130)	n (%)		60 (63.2%)	236 (65.9%)	130 (49.4%)	Chi-square test	< 0.001	0.001
Hematocrit	526 (79, 307, 140)	Median (IQR)	%	39.70 (32.45, 46.65)	36.00 (28.45, 44.40)	33.90 (27.05, 38.78)	Kruskal-Wallis test	< 0.001	0.001
PLA2R	54 (14, 33, 7)	n (%)		14 (14.7%)	33 (9.2%)	7 (2.7%)	Chi-square test	< 0.001	0.001
Hyperphosphatemia at Admission	142 (14, 93, 35)	n (%)		14 (14.7%)	93 (26.0%)	35 (13.3%)	Chi-square test	< 0.001	0.001
IBIL	245 (42, 133, 70)	Median (IQR)	μmol/L	3.40 (1.42, 4.97)	2.40 (1.60, 4.30)	4.05 (2.52, 6.77)	Kruskal-Wallis test	< 0.001	0.002
Urine Granular Casts_Microscopic	97 (20, 53, 24)	Median (IQR)	count per HPF	0.50 (0.00, 7.00)	0.00 (0.00, 3.00)	0.00 (0.00, 0.00)	Kruskal-Wallis test	< 0.001	0.002
Pneumonia	546 (59, 271, 216)	n (%)		59 (62.1%)	271 (75.7%)	216 (82.1%)	Chi-square test	< 0.001	0.002
Lipase	310 (48, 203, 59)	Median (IQR)	U/L	28.80 (21.25, 38.10)	29.00 (19.15, 42.70)	37.00 (27.90, 60.50)	Kruskal-Wallis test	< 0.001	0.002
HGB	526 (79, 307, 140)	Median (IQR)	g/L	127.00 (104.00, 152.00)	117.00 (92.00, 146.00)	108.50 (88.75, 129.00)	Kruskal-Wallis test	0.001	0.003

Characteristic	N (Riva, Indo, Clop)	Data Type	Unit	Rivaroxaban	Indobufen	Clopidogrel	Statistical Method	P-value	FDR P-value
Creatinine	514 (82, 304, 128)	Median (IQR)	μmol/L	94.00 (70.03, 142.43)	108.50 (72.45, 224.00)	143.05 (90.60, 350.05)	Kruskal-Wallis test	0.001	0.003
Facial Edema	185 (18, 116, 51)	n (%)		18 (18.9%)	115 (32.1%)	51 (19.4%)	Fisher's exact test (with simulated p-value)	0.001	0.003
Cholinesterase	245 (42, 133, 70)	Median (IQR)	U/L	8645.50 (7187.25, 11429.25)	9424.00 (6806.00, 12290.00)	7568.50 (5484.50, 9588.50)	Kruskal-Wallis test	0.001	0.004
Adenosine Deaminase	246 (42, 133, 71)	Median (IQR)	U/L	10.00 (7.58, 14.78)	13.50 (10.00, 19.50)	11.10 (7.20, 15.80)	Kruskal-Wallis test	0.001	0.004
Diabetes Mellitus	137 (16, 52, 69)	n (%)		16 (16.8%)	52 (14.5%)	69 (26.2%)	Chi-square test	0.001	0.005
Diffuse Renal Disease	133 (18, 84, 31)	n (%)		18 (18.9%)	84 (23.5%)	31 (11.8%)	Chi-square test	0.001	0.005
Sodium	548 (87, 318, 143)	Median (IQR)	mmol/L	141.00 (139.00, 143.00)	140.00 (137.53, 142.00)	141.40 (138.80, 143.00)	Kruskal-Wallis test	0.001	0.005
Bronchitis	136 (21, 49, 66)	n (%)		21 (22.1%)	49 (13.7%)	66 (25.1%)	Chi-square test	0.001	0.005

Characteristic	N (Riva, Indo, Clop)	Data Type	Unit	Rivaroxaban	Indobufen	Clopidogrel	Statistical Method	P-value	FDR P-value
Triglycerides at Admission	558 (110, 306, 142)	[Normal, Mildly elevated, Moderately elevated, Severely elevated]		44(40.0%), 19(17.3%), 40(36.4%), 7(6.4%)	116(37.9%), 58(19.0%), 118(38.6%), 14(4.6%)	79(55.6%), 22(15.5%), 38(26.8%), 3(2.1%)	Kruskal-Wallis test (for ordered data)	0.001	0.008
PDW	514 (75, 301, 138)	Median (IQR)	%	12.20 (9.85, 15.75)	10.40 (8.90, 12.80)	10.55 (9.80, 12.20)	Kruskal-Wallis test	0.002	0.009
Hypertension	425 (50, 197, 178)	n (%)		50 (52.6%)	197 (55.0%)	178 (67.7%)	Chi-square test	0.002	0.009
Gender	716 (132, 411, 231)	[Female, Male]		59 (62.1%)	244 (68.2%)	144 (54.8%)	Chi-square test	0.003	0.012
Antithrombin III	304 (59, 192, 53)	Mean ± SD	% activity	74.59 ± 24.84	75.10 ± 25.53	87.73 ± 22.18	One-way ANOVA	0.004	0.014
Hyponatremia at Admission	45 (5, 33, 7)	n (%)		5 (5.3%)	33 (9.2%)	7 (2.7%)	Chi-square test	0.004	0.014
TT	489 (73, 285, 131)	Median (IQR)	seconds	17.70 (16.60, 19.10)	18.40 (16.70, 20.70)	18.90 (17.20, 20.25)	Kruskal-Wallis test	0.004	0.016

Characteristic	N (Riva, Indo, Clop)	Data Type	Unit	Rivaroxaban	Indobufen	Clopidogrel	Statistical Method	P-value	FDR P-value
WBC	526 (79, 307, 140)	Median (IQR)	10^9/L	8.08 (6.28, 11.45)	8.39 (6.70, 11.50)	7.28 (5.86, 9.51)	Kruskal-Wallis test	0.006	0.021
Minimal Change Disease (MCD)	62 (5, 43, 14)	n (%)		5 (5.3%)	43 (12.0%)	14 (5.3%)	Chi-square test	0.006	0.022
TBIL	245 (42, 133, 70)	Median (IQR)	μmol/L	5.15 (2.73, 6.68)	4.00 (2.90, 6.30)	5.85 (3.70, 9.25)	Kruskal-Wallis test	0.007	0.023
Globulin	245 (42, 133, 70)	Median (IQR)	g/L	22.40 (20.57, 24.85)	23.50 (20.60, 27.10)	25.55 (22.30, 29.65)	Kruskal-Wallis test	0.007	0.025
Hypothyroidism	129 (21, 76, 32)	n (%)		21 (22.1%)	76 (21.2%)	32 (12.2%)	Chi-square test	0.008	0.027
Emphysema	114 (14, 44, 56)	n (%)		14 (14.7%)	44 (12.3%)	56 (21.3%)	Chi-square test	0.010	0.032
Blood Urea Nitrogen	514 (82, 304, 128)	Median (IQR)	mmol/L	7.86 (5.00, 12.23)	8.44 (5.68, 15.21)	10.00 (6.14, 16.62)	Kruskal-Wallis test	0.010	0.035
Lupus Nephritis (LN)	106 (6, 51, 49)	n (%)		6 (6.3%)	51 (14.2%)	49 (18.6%)	Chi-square test	0.014	0.045
Basophil Percentage	526 (79, 307, 140)	Median (IQR)	%	0.30 (0.20, 0.50)	0.30 (0.20, 0.50)	0.40 (0.20, 0.60)	Kruskal-Wallis test	0.016	0.049

Characteristic	N (Riva, Indo, Clop)	Data Type	Unit	Rivaroxaban	Indobufen	Clopidogrel	Statistical Method	P-value	FDR P-value
FIB	489 (73, 285, 131)	Median (IQR)	g/L	4.75 (3.70, 6.60)	4.92 (3.89, 6.58)	4.32 (3.33, 5.89)	Kruskal-Wallis test	0.018	0.057
Absolute Iron Deficiency at Admission	7 (3, 4, 0)	n (%)		3 (3.2%)	4 (1.1%)	0 (0.0%)	Fisher's exact test (with simulated p-value)	0.019	0.057
Prothrombin Time Ratio	489 (73, 285, 131)	Median (IQR)	ratio	0.93 (0.87, 0.99)	0.95 (0.88, 1.01)	0.96 (0.91, 1.01)	Kruskal-Wallis test	0.020	0.060
Absolute Lymphocyte Count	526 (79, 307, 140)	Median (IQR)	10^9/L	1.63 (1.17, 2.23)	1.52 (0.95, 2.21)	1.40 (0.81, 1.88)	Kruskal-Wallis test	0.021	0.061
Gastric Ulcer	4 (2, 0, 2)	n (%)		2 (2.1%)	0 (0.0%)	2 (0.8%)	Fisher's exact test (with simulated p-value)	0.021	0.062
PLT	526 (79, 307, 140)	Median (IQR)	10^9/L	285.00 (238.00, 334.50)	279.00 (218.50, 352.50)	252.00 (198.50, 322.50)	Kruskal-Wallis test	0.022	0.064
Hypomagnesemia at Admission	139 (14, 84, 41)	n (%)		14 (14.7%)	84 (23.5%)	41 (15.6%)	Chi-square test	0.023	0.066

Characteristic	N (Riva, Indo, Clop)	Data Type	Unit	Rivaroxaban	Indobufen	Clopidogrel	Statistical Method	P-value	FDR P-value
Anemia	300 (28, 158, 114)	n (%)		28 (29.5%)	158 (44.1%)	114 (43.3%)	Chi-square test	0.030	0.084
International Normalized Ratio	489 (73, 285, 131)	Median (IQR)	ratio	0.92 (0.87, 1.00)	0.94 (0.88, 1.01)	0.94 (0.91, 1.01)	Kruskal-Wallis test	0.035	0.094
Inorganic Phosphorus	542 (86, 315, 141)	Median (IQR)	mmol/L	1.19 (1.07, 1.36)	1.27 (1.09, 1.47)	1.18 (1.05, 1.39)	Kruskal-Wallis test	0.044	0.115
Absolute Neutrophil Count	526 (79, 307, 140)	Median (IQR)	10^9/L	5.46 (3.86, 8.51)	5.89 (4.29, 9.06)	4.92 (3.76, 7.49)	Kruskal-Wallis test	0.045	0.116
Thyroid Nodule	103 (14, 62, 27)	n (%)		14 (14.7%)	62 (17.3%)	27 (10.3%)	Chi-square test	0.047	0.119
Urine Red Blood Cells_Microscopic	198 (29, 107, 62)	Median (IQR)	cells/HPF	1.50 (0.20, 8.00)	4.00 (1.00, 12.25)	2.00 (0.00, 9.75)	Kruskal-Wallis test	0.052	0.131
eGFR	427 (54, 261, 112)	Median (IQR)	mL/min/1.73 m^2	45.84 (25.31, 62.69)	38.70 (18.27, 65.91)	30.21 (13.47, 58.54)	Kruskal-Wallis test	0.053	0.131
Cystatin C	427 (54, 261, 112)	Median (IQR)	mg/L	1.44 (1.14, 2.25)	1.64 (1.10, 2.88)	1.98 (1.20, 3.62)	Kruskal-Wallis test	0.053	0.131
Alkaline Phosphatase	245 (42, 133, 70)	Median (IQR)	U/L	65.50 (53.50, 88.00)	75.00 (61.00, 91.00)	73.00 (59.25, 102.50)	Kruskal-Wallis test	0.053	0.131

Characteristic	N (Riva, Indo, Clop)	Data Type	Unit	Rivaroxaban	Indobufen	Clopidogrel	Statistical Method	P-value	FDR P-value
Hyperparathyroidism	55 (4, 36, 15)	n (%)		4 (4.2%)	36 (10.1%)	15 (5.7%)	Fisher's exact test (with simulated p-value)	0.055	0.134
Upper Respiratory Tract Infection (URTI)	20 (4, 13, 3)	n (%)		4 (4.2%)	13 (3.6%)	3 (1.1%)	Fisher's exact test (with simulated p-value)	0.068	0.162
High_Density Lipoprotein Cholesterol	162 (26, 97, 39)	Median (IQR)	mmol/L	1.40 (1.07, 2.01)	1.56 (1.26, 2.10)	1.28 (0.98, 1.82)	Kruskal-Wallis test	0.069	0.162
Low Hemoglobin at Admission	251 (31, 140, 80)	n (%)		31 (32.6%)	140 (39.1%)	80 (30.4%)	Chi-square test	0.070	0.164
Urine White Blood Cells_Microscopic	198 (29, 107, 62)	Median (IQR)	cells/HPF	3.00 (0.60, 5.00)	5.00 (1.80, 13.10)	2.70 (0.60, 8.93)	Kruskal-Wallis test	0.074	0.170
Urine Other Casts_Microscopic	198 (29, 107, 62)	Median (IQR)	cells/HPF	0.00 (0.00, 0.00)	0.00 (0.00, 0.00)	0.00 (0.00, 0.00)	Kruskal-Wallis test	0.075	0.170

Characteristic	N (Riva, Indo, Clop)	Data Type	Unit	Rivaroxaban	Indobufen	Clopidogrel	Statistical Method	P-value	FDR P-value
Bleeding Tendency at Admission	30 (3, 21, 6)	n (%)		3 (3.2%)	21 (5.9%)	6 (2.3%)	Fisher's exact test (with simulated p-value)	0.085	0.190
Potassium	548 (87, 318, 143)	Median (IQR)	mmol/L	3.94 (3.70, 4.31)	4.15 (3.76, 4.60)	4.07 (3.64, 4.62)	Kruskal-Wallis test	0.088	0.195
FDP	304 (59, 192, 53)	Median (IQR)	mg/L	3.43 (1.55, 6.08)	4.23 (2.40, 7.95)	4.70 (2.93, 8.38)	Kruskal-Wallis test	0.089	0.197
Membranous Nephropathy (MN)	183 (31, 81, 71)	n (%)		31 (32.6%)	81 (22.6%)	71 (27.0%)	Chi-square test	0.111	0.234
APTT	489 (73, 285, 131)	Median (IQR)	seconds	30.80 (27.40, 35.40)	33.60 (28.20, 39.40)	32.60 (28.70, 38.20)	Kruskal-Wallis test	0.116	0.243
Rheumatoid Arthritis (RA)	9 (1, 2, 6)	n (%)		1 (1.1%)	2 (0.6%)	6 (2.3%)	Fisher's exact test (with simulated p-value)	0.120	0.251
MCHC	526 (79, 307, 140)	Median (IQR)	g/L	324.00 (315.00, 334.50)	328.00 (317.50, 337.00)	326.50 (317.00, 334.00)	Kruskal-Wallis test	0.126	0.262

Characteristic	N (Riva, Indo, Clop)	Data Type	Unit	Rivaroxaban	Indobufen	Clopidogrel	Statistical Method	P-value	FDR P-value
Urine Other Crystals_Microscopic	97 (20, 53, 24)	Median (IQR)	cells/HPF	0.00 (0.00, 0.00)	0.00 (0.00, 0.00)	0.00 (0.00, 0.00)	Kruskal-Wallis test	0.193	0.361
Hypokalemia at Admission	79 (13, 44, 22)	n (%)		13 (13.7%)	44 (12.3%)	22 (8.4%)	Chi-square test	0.206	0.378
Triglycerides	165 (28, 97, 40)	Median (IQR)	mmol/L	2.37 (1.53, 3.17)	2.38 (1.59, 3.40)	1.88 (1.39, 2.79)	Kruskal-Wallis test	0.213	0.386
Urine Hyaline Casts_Microscopic	97 (20, 53, 24)	Median (IQR)	cells/HPF	2.00 (0.00, 4.25)	1.00 (0.00, 5.00)	0.00 (0.00, 2.00)	Kruskal-Wallis test	0.225	0.403
Plateletcrit	514 (75, 301, 138)	Median (IQR)	%	0.26 (0.22, 0.32)	0.26 (0.20, 0.32)	0.24 (0.19, 0.32)	Kruskal-Wallis test	0.226	0.403
Hyperkalemia at Admission	18 (0, 11, 7)	n (%)		0 (0.0%)	11 (3.1%)	7 (2.7%)	Fisher's exact test (with simulated p-value)	0.238	0.414
Amylase	312 (49, 203, 60)	Median (IQR)	U/L	68.00 (52.00, 90.00)	63.00 (45.00, 89.50)	71.00 (53.25, 106.00)	Kruskal-Wallis test	0.238	0.414
Uric Acid	535 (84, 312, 139)	Median (IQR)	µmol/L	388.90 (320.60, 494.05)	432.80 (343.23, 522.52)	423.40 (329.55, 510.75)	Kruskal-Wallis test	0.254	0.435

Characteristic	N (Riva, Indo, Clop)	Data Type	Unit	Rivaroxaban	Indobufen	Clopidogrel	Statistical Method	P-value	FDR P-value
Total Bile Acid	245 (42, 133, 70)	Median (IQR)	μmol/L	2.15 (1.22, 6.25)	2.10 (1.00, 4.80)	2.40 (1.50, 4.85)	Kruskal-Wallis test	0.260	0.439
Urine Dipstick Glucose at Admission	514 (102, 274, 138)	[Negative, Trace, Positive, Strong Positive]		65(63.7%), 13(12.7%), 11(10.8%), 13(12.7%)	195(71.2%), 28(10.2%), 28(10.2%), 23(8.4%)	100(72.5%), 17(12.3%), 3(2.2%), 18(13.0%)	Kruskal-Wallis test (for ordered data)	0.288	0.457
Eosinophil Percentage	526 (79, 307, 140)	Median (IQR)	%	1.50 (0.40, 3.90)	1.00 (0.20, 3.00)	1.20 (0.40, 3.00)	Kruskal-Wallis test	0.294	0.485
Aspartate Aminotransferase	246 (42, 134, 70)	Median (IQR)	U/L	18.00 (14.00, 24.75)	20.00 (15.25, 28.75)	20.00 (15.00, 24.75)	Kruskal-Wallis test	0.303	0.494
RDW-CV	526 (79, 307, 140)	Median (IQR)	%	13.60 (12.80, 14.95)	14.00 (12.90, 15.30)	14.10 (13.07, 15.53)	Kruskal-Wallis test	0.304	0.494
Absolute Monocyte Count	526 (79, 307, 140)	Median (IQR)	10^9/L	0.55 (0.38, 0.70)	0.51 (0.38, 0.71)	0.48 (0.37, 0.64)	Kruskal-Wallis test	0.322	0.512
Creatine Kinase	450 (66, 265, 119)	Median (IQR)	U/L	117.00 (70.00, 177.00)	122.00 (65.00, 211.00)	144.00 (75.50, 239.50)	Kruskal-Wallis test	0.328	0.516
Absolute Eosinophil Count	526 (79, 307, 140)	Median (IQR)	10^9/L	0.13 (0.04, 0.25)	0.09 (0.02, 0.22)	0.10 (0.03, 0.19)	Kruskal-Wallis test	0.363	0.558

Characteristic	N (Riva, Indo, Clop)	Data Type	Unit	Rivaroxaban	Indobufen	Clopidogrel	Statistical Method	P-value	FDR P-value
Absolute Basophil Count	526 (79, 307, 140)	Median (IQR)	10^9/L	0.03 (0.01, 0.05)	0.03 (0.01, 0.04)	0.03 (0.02, 0.05)	Kruskal-Wallis test	0.363	0.558
Bicarbonate	538 (87, 312, 139)	Median (IQR)	mmol/L	23.80 (21.10, 26.10)	23.10 (19.90, 26.00)	22.90 (18.75, 26.05)	Kruskal-Wallis test	0.382	0.584
MCV	526 (79, 307, 140)	Median (IQR)	fL	87.00 (78.75, 90.80)	87.40 (84.00, 90.60)	87.95 (83.83, 91.88)	Kruskal-Wallis test	0.384	0.586
D-Dimer	428 (68, 253, 107)	Median (IQR)	mg/L or µg/mL	1.14 (0.47, 2.25)	1.33 (0.80, 2.54)	1.28 (0.66, 2.39)	Kruskal-Wallis test	0.398	0.598
Lymphocyte Percentage	526 (79, 307, 140)	Median (IQR)	%	22.30 (11.60, 26.85)	17.10 (11.20, 26.85)	18.65 (11.38, 26.82)	Kruskal-Wallis test	0.399	0.598
Prealbumin	240 (41, 130, 69)	Median (IQR)	mg/L	241.00 (158.00, 321.00)	215.30 (151.12, 303.17)	248.00 (195.50, 294.90)	Kruskal-Wallis test	0.413	0.611
Magnesium	542 (86, 315, 141)	Median (IQR)	mmol/L	0.87 (0.77, 0.92)	0.83 (0.76, 0.92)	0.82 (0.74, 0.93)	Kruskal-Wallis test	0.429	0.625
UPCR at Admission	137 (57, 72, 8)	[Normal, Mildly elevated, Severely elevated]		27(47.4%), 8(14.0%), 22(38.6%)	31(43.1%), 10(13.9%), 31(43.1%)	5(62.5%), 1(12.5%), 2(25.0%)	Kruskal-Wallis test (for ordered data)	0.530	0.685

Characteristic	N (Riva, Indo, Clop)	Data Type	Unit	Rivaroxaban	Indobufen	Clopidogrel	Statistical Method	P-value	FDR P-value
Alanine Aminotransferase	246 (42, 134, 70)	Median (IQR)	U/L	19.50 (15.00, 26.75)	16.00 (12.00, 27.75)	17.00 (12.00, 24.75)	Kruskal-Wallis test	0.494	0.699
Immunoglobulin G	138 (27, 80, 31)	Median (IQR)	g/L	4.41 (3.31, 6.10)	5.25 (3.38, 7.76)	5.53 (3.76, 7.62)	Kruskal-Wallis test	0.509	0.705
Urine Dipstick Leukocytes at Admission	215 (42, 111, 62)	[Negative, Trace, Positive, Strong Positive]		36(85.7%), 3(7.1%), 2(4.8%), 1(2.4%)	96(86.5%), 8(7.2%), 2(1.8%), 5(4.5%)	57(91.9%), 2(3.2%), 0(0.0%),3(4.8%)	Kruskal-Wallis test (for ordered data)	0.564	0.714
Neutrophil Percentage	526 (79, 307, 140)	Median (IQR)	%	68.50 (61.55, 78.40)	71.20 (61.25, 81.90)	71.00 (61.60, 80.75)	Kruskal-Wallis test	0.532	0.734
Hypernatremia at Admission	46 (8, 20, 18)	n (%)		8 (8.4%)	20 (5.6%)	18 (6.8%)	Chi-square test	0.570	0.771
UACR at Admission	221 (28, 164, 29)	[Normoalbuminuria, Microalbuminuria, Macroalbuminuria]		1(3.6%), 1(3.6%), 26(92.9%)	2(1.2%), 4(2.4%), 158(96.3%)	0(0.0%), 1(3.4%), 28(96.6%)	Kruskal-Wallis test (for ordered data)	0.671	0.799
MCH	526 (79, 307, 140)	Median (IQR)	pg	28.70 (25.55, 30.10)	28.90 (27.40, 30.00)	28.80 (27.20, 30.00)	Kruskal-Wallis test	0.605	0.812

Characteristic	N (Riva, Indo, Clop)	Data Type	Unit	Rivaroxaban	Indobufen	Clopidogrel	Statistical Method	P-value	FDR P-value
Thrombocytopenia	13 (2, 8, 3)	n (%)		2 (2.1%)	8 (2.2%)	3 (1.1%)	Fisher's exact test (with simulated p-value)	0.621	0.829
Monocyte Percentage	526 (79, 307, 140)	Median (IQR)	%	6.20 (5.00, 8.00)	6.40 (4.50, 8.15)	6.85 (5.10, 8.20)	Kruskal-Wallis test	0.638	0.845
Hypermagnesemia at Admission	2 (0, 2, 0)	n (%)		0 (0.0%)	2 (0.6%)	0 (0.0%)	Fisher's exact test (with simulated p-value)	0.641	0.845
Abdominal Wall Edema	45 (4, 25, 16)	n (%)		4 (4.2%)	25 (7.0%)	16 (6.1%)	Fisher's exact test (with simulated p-value)	0.646	0.848
G6PD Deficiency	43 (6, 24, 13)	n (%)		6 (6.3%)	24 (6.7%)	13 (4.9%)	Chi-square test	0.653	0.854
DBIL	245 (42, 133, 70)	Median (IQR)	μmol/L	1.65 (1.40, 2.00)	1.50 (1.10, 2.00)	1.45 (1.00, 2.68)	Kruskal-Wallis test	0.659	0.857

Characteristic	N (Riva, Indo, Clop)	Data Type	Unit	Rivaroxaban	Indobufen	Clopidogrel	Statistical Method	P-value	FDR P-value
IgA Nephropathy (IgAN)	18 (3, 10, 5)	n (%)		3 (3.2%)	10 (2.8%)	5 (1.9%)	Fisher's exact test (with simulated p-value)	0.681	0.882
Functional Iron Deficiency at Admission	3 (0, 1, 2)	n (%)		0 (0.0%)	1 (0.3%)	2 (0.8%)	Fisher's exact test (with simulated p-value)	0.728	0.930
HBDH	414 (65, 240, 109)	Median (IQR)	U/L	179.00 (144.00, 215.00)	183.50 (145.90, 234.50)	179.00 (150.00, 226.00)	Kruskal-Wallis test	0.757	0.951
Hypophosphatemia at Admission	28 (5, 13, 10)	n (%)		5 (5.3%)	13 (3.6%)	10 (3.8%)	Chi-square test	0.762	0.952
Gamma-Glutamyl Transferase	245 (42, 133, 70)	Median (IQR)	U/L	31.50 (21.00, 43.75)	26.00 (18.00, 47.00)	28.50 (17.00, 57.00)	Kruskal-Wallis test	0.792	0.982
Chloride	548 (87, 318, 143)	Mean ± SD	mmol/L	108.37 ± 5.26	108.16 ± 5.59	108.51 ± 5.99	One-way ANOVA	0.810	0.997
LDH	450 (66, 265, 119)	Median (IQR)	U/L	238.50 (202.25, 286.50)	243.00 (204.00, 308.00)	240.00 (205.00, 302.00)	Kruskal-Wallis test	0.815	0.998

Characteristic	N (Riva, Indo, Clop)	Data Type	Unit	Rivaroxaban	Indobufen	Clopidogrel	Statistical Method	P-value	FDR P-value
Prothrombin Activity	489 (73, 285, 131)	Median (IQR)	%	104.80 (95.20, 113.10)	103.80 (93.00, 114.60)	103.00 (93.00, 115.00)	Kruskal-Wallis test	0.951	1.000

Data are presented as median (interquartile range) for continuous variables and n (%) for categorical variables. The total number of patients with available data for each variable is shown, and percentages are calculated based on these numbers. P-values were calculated using the Kruskal-Wallis test for continuous variables and the Chi-squared test or Fisher's exact test for categorical variables, as appropriate. P-values were adjusted for multiple comparisons using the False Discovery Rate (FDR) method.

Abbreviations: FDR, False Discovery Rate.

Supplementary Table S2. Comprehensive Comparison of All Measured Laboratory Parameters at Baseline and Follow-up.

**Characteristic**	**Rivaroxaban_n**	**Rivaroxaban_Baseline Mean**	**Rivaroxaban_Follow-up Mean**	**Rivaroxaban_Mean Difference**	**Rivaroxaban_P value**	**Indobufen_n**	**Indobufen_Baseline Mean**	**Indobufen_Follow-up Mean**	**Indobufen_Mean Difference**	**Indobufen_P value**
Urine Protein	28	2.57	1.23	-1.34	< 0.001	78	2.31	1.02	-1.30	< 0.001
Low_Density Lipoprotein Cholesterol	48	6.84	5.39	-1.45	< 0.001	135	6.24	4.56	-1.67	< 0.001
High_Density Lipoprotein Cholesterol	48	1.42	1.98	0.56	< 0.001	133	1.48	2.06	0.58	< 0.001
MPV	55	9.40	8.95	-0.45	< 0.001	181	9.45	8.96	-0.49	< 0.001
Prealbumin	65	185.93	327.37	141.45	< 0.001	176	216.15	353.88	137.73	< 0.001
DBIL	64	1.38	2.28	0.90	< 0.001	172	1.77	2.38	0.61	< 0.001
Albumin/Globulin Ratio	66	0.86	1.06	0.21	< 0.001	179	0.99	1.10	0.11	< 0.001

Characteristic	Rivaroxaban_n	Rivaroxaban_Baseline Mean	Rivaroxaban_Follow-up Mean	Rivaroxaban_Mean Difference	Rivaroxaban_P value	Indobufen_n	Indobufen_Baseline Mean	Indobufen_Follow-up Mean	Indobufen_Mean Difference	Indobufen_P value
Total Bile Acid	68	2.62	4.43	1.82	< 0.001	181	4.48	5.54	1.07	0.263
Globulin	65	21.84	24.06	2.22	< 0.001	182	21.69	24.84	3.15	< 0.001
Total Protein	68	40.03	48.24	8.20	< 0.001	179	42.09	50.75	8.66	< 0.001
ALB	68	18.15	24.94	6.79	< 0.001	179	20.37	26.50	6.13	< 0.001
White Blood Cell Count	66	10.42	12.97	2.55	< 0.001	189	9.83	12.38	2.55	< 0.001
Basophil Percentage	54	0.37	0.22	-0.16	< 0.001	153	0.44	0.31	-0.14	< 0.001
RDW-CV	61	14.44	15.00	0.56	< 0.001	175	14.29	14.79	0.50	< 0.001
Calcium	69	1.88	2.05	0.17	< 0.001	204	1.92	2.10	0.17	< 0.001

Characteristic	Rivaroxaban_n	Rivaroxaban_Baseline Mean	Rivaroxaban_Follow-up Mean	Rivaroxaban_Mean Difference	Rivaroxaban_P value	Indobufen_n	Indobufen_Baseline Mean	Indobufen_Follow-up Mean	Indobufen_Mean Difference	Indobufen_P value
Urine Protein Concentration	35	10.17	4.15	-6.02	0.001	77	8.87	4.81	-4.06	< 0.001
Urine Occult Blood	36	1.35	0.77	-0.58	0.001	104	1.29	0.44	-0.85	< 0.001
TBIL	67	4.29	6.59	2.30	0.001	178	5.20	6.79	1.60	< 0.001
Absolute Neutrophil Count	66	7.61	9.85	2.24	0.001	187	7.14	9.04	1.89	< 0.001
Chloride	69	109.00	107.01	-1.99	0.001	212	108.79	106.90	-1.89	< 0.001
Immunoglobulin G	38	4.08	5.05	0.98	0.002	71	5.42	5.63	0.21	0.527
Total Cholesterol	51	10.30	8.89	-1.41	0.003	140	9.14	7.70	-1.44	< 0.001
PDW	61	12.72	14.22	1.49	0.003	179	11.05	13.64	2.59	< 0.001

Characteristic	Rivaroxaban_n	Rivaroxaban_Baseline Mean	Rivaroxaban_Follow-up Mean	Rivaroxaban_Mean Difference	Rivaroxaban_P value	Indobufen_n	Indobufen_Baseline Mean	Indobufen_Follow-up Mean	Indobufen_Mean Difference	Indobufen_P value
Cholinesterase	67	8777.13	7925.39	-851.75	0.003	179	9834.53	8539.66	-1294.87	< 0.001
IBIL	66	2.83	4.24	1.41	0.004	177	3.43	4.44	1.00	< 0.001
Leucine Aminopeptidase	65	45.65	52.14	6.50	0.005	176	39.20	47.93	8.74	< 0.001
Bicarbonate	68	23.41	24.59	1.18	0.007	203	23.76	24.57	0.81	0.011
FIB	20	5.42	3.95	-1.48	0.008	62	5.24	4.19	-1.05	0.001
Neutrophil Percentage	66	69.59	74.51	4.92	0.008	187	69.66	71.78	2.13	0.069
Uric Acid	71	380.26	344.52	-35.74	0.011	208	413.33	372.33	-41.00	< 0.001
Glomerular Filtration Rate	44	43.32	49.28	5.96	0.014	172	46.10	42.76	-3.34	0.016

Characteristic	Rivaroxaban_n	Rivaroxaban_Baseline Mean	Rivaroxaban_Follow-up Mean	Rivaroxaban_Mean Difference	Rivaroxaban_P value	Indobufen_n	Indobufen_Baseline Mean	Indobufen_Follow-up Mean	Indobufen_Mean Difference	Indobufen_P value
MCV	64	84.57	85.15	0.58	0.018	184	84.88	85.68	0.80	< 0.001
Lymphocyte Percentage	66	21.88	18.21	-3.67	0.020	186	21.01	20.16	-0.85	0.365
Antithrombin III	9	55.88	87.18	31.30	0.026	38	74.64	90.29	15.64	0.004
Urine Granular Casts_Microscopic	6	6.50	0.83	-5.67	0.032	21	2.43	0.52	-1.91	0.016
T4	8	39.32	62.27	22.95	0.034	41	59.57	83.65	24.08	< 0.001
Creatinine	70	134.90	117.58	-17.32	0.035	199	175.90	179.28	3.38	0.711
HBDH	12	218.13	247.45	29.33	0.037	36	273.13	261.83	-11.30	0.651
Complement C1q	12	16.38	14.27	-2.11	0.040	34	16.77	22.32	5.55	0.234

Characteristic	Rivaroxaban_n	Rivaroxaban_Baseline Mean	Rivaroxaban_Follow-up Mean	Rivaroxaban_Mean Difference	Rivaroxaban_P value	Indobufen_n	Indobufen_Baseline Mean	Indobufen_Follow-up Mean	Indobufen_Mean Difference	Indobufen_P value
Adenosine Deaminase	66	13.78	12.68	-1.10	0.040	178	14.66	12.99	-1.67	< 0.001
D-Dimer	17	3.26	2.20	-1.06	0.043	51	2.36	3.22	0.86	0.101
24_hour Urine Volume	20	1360.50	1750.00	389.50	0.043	58	1324.83	1471.03	146.21	0.129
MCHC	58	321.95	319.28	-2.67	0.043	183	326.28	325.65	-0.64	0.466
FT3	8	2.21	3.42	1.21	0.044	41	3.39	4.14	0.75	0.069
Cystatin C	44	1.83	1.62	-0.21	0.046	171	2.09	2.19	0.10	0.110
Eosinophil Percentage	58	1.81	1.25	-0.56	0.050	177	2.46	1.51	-0.95	0.001
Absolute Monocyte Count	66	0.64	0.75	0.11	0.051	184	0.62	0.76	0.13	< 0.001

Characteristic	Rivaroxaban_n	Rivaroxaban_Baseline Mean	Rivaroxaban_Follow-up Mean	Rivaroxaban_Mean Difference	Rivaroxaban_P value	Indobufen_n	Indobufen_Baseline Mean	Indobufen_Follow-up Mean	Indobufen_Mean Difference	Indobufen_P value
Absolute Basophil Count	54	0.03	0.03	-0.01	0.053	153	0.04	0.03	-0.01	0.027
Monocyte Percentage	65	6.56	5.82	-0.74	0.063	184	6.66	6.32	-0.34	0.187
ApoA1	12	1.28	1.59	0.30	0.064	95	1.46	1.78	0.32	< 0.001
FT4	8	7.94	10.47	2.53	0.065	41	12.04	16.79	4.75	0.001
T3	8	0.91	1.28	0.38	0.066	41	1.19	1.40	0.21	0.036
Lipoprotein a	20	1081.50	715.52	-365.98	0.067	98	916.56	595.52	-321.04	< 0.001
Hematocrit	66	37.19	38.26	1.08	0.067	188	37.07	38.13	1.06	0.003
Apolipoprotein B	21	1.94	1.61	-0.33	0.077	103	1.66	1.31	-0.35	< 0.001

Characteristic	Rivaroxaban_n	Rivaroxaban_Baseline Mean	Rivaroxaban_Follow-up Mean	Rivaroxaban_Mean Difference	Rivaroxaban_P value	Indobufen_n	Indobufen_Baseline Mean	Indobufen_Follow-up Mean	Indobufen_Mean Difference	Indobufen_P value
Inorganic Phosphorus	70	1.20	1.14	-0.06	0.079	201	1.31	1.21	-0.10	< 0.001
Urine Vitamin C	7	1.57	0.44	-1.13	0.090	14	1.10	1.28	0.18	0.866
Aspartate Aminotransferase	65	27.40	21.85	-5.55	0.096	171	30.67	23.09	-7.58	0.033
FDP	9	4.59	3.60	-1.00	0.106	38	6.36	8.92	2.56	0.201
Urine Bacteria_Microscopic	39	129.77	9.21	-120.56	0.108	120	45.63	19.75	-25.88	0.124
Urine Hyaline Casts_Microscopic	7	3.14	0.64	-2.50	0.113	34	3.53	1.03	-2.50	0.001
Urine Other Casts_Microscopic	8	17.75	1.38	-16.38	0.114	31	14.94	5.73	-9.21	0.021

Characteristic	Rivaroxaban_n	Rivaroxaban_Baseline Mean	Rivaroxaban_Follow-up Mean	Rivaroxaban_Mean Difference	Rivaroxaban_P value	Indobufen_n	Indobufen_Baseline Mean	Indobufen_Follow-up Mean	Indobufen_Mean Difference	Indobufen_P value
24_hour Urine Protein	20	7.42	5.44	-1.99	0.117	56	7.07	6.02	-1.04	0.153
UACR	2	5885.17	3693.13	-2192.04	0.119	18	4689.55	4725.74	36.19	0.979
PT	19	11.83	12.75	0.91	0.125	61	11.43	11.47	0.04	0.890
Iron	4	6.07	9.25	3.18	0.137	19	10.80	8.49	-2.31	0.243
Magnesium	68	0.84	0.81	-0.03	0.151	197	0.81	0.82	0.01	0.324
Red Blood Cell Count	65	4.43	4.53	0.10	0.186	187	4.40	4.49	0.09	0.026
Immunoglobulin M	15	1.39	1.30	-0.09	0.201	43	2.19	1.61	-0.58	0.192
HGB	65	121.34	123.80	2.46	0.210	182	121.36	124.85	3.49	0.004

Characteristic	Rivaroxaban_n	Rivaroxaban_Baseline Mean	Rivaroxaban_Follow-up Mean	Rivaroxaban_Mean Difference	Rivaroxaban_P value	Indobufen_n	Indobufen_Baseline Mean	Indobufen_Follow-up Mean	Indobufen_Mean Difference	Indobufen_P value
TSH	8	18.81	9.14	-9.67	0.220	40	5.70	3.35	-2.35	0.002
International Normalized Ratio	20	1.02	1.08	0.06	0.247	58	0.95	0.96	0.01	0.590
Immunoglobulin E	14	296.21	360.43	64.21	0.248	41	593.46	411.13	-182.33	0.202
Absolute Lymphocyte Count	66	2.02	2.22	0.20	0.256	186	1.87	2.43	0.57	< 0.001
Prothrombin Time Ratio	20	1.02	1.07	0.05	0.262	59	0.94	0.95	0.01	0.716
Urine Nitrite	5	0.80	0.30	-0.50	0.266	21	0.24	0.67	0.43	0.023
Urine White Blood Cells_Microscopic	51	11.35	8.24	-3.11	0.286	149	10.05	16.04	5.99	0.346

Characteristic	Rivaroxaban_n	Rivaroxaban_Baseline Mean	Rivaroxaban_Follow-up Mean	Rivaroxaban_Mean Difference	Rivaroxaban_P value	Indobufen_n	Indobufen_Baseline Mean	Indobufen_Follow-up Mean	Indobufen_Mean Difference	Indobufen_P value
TT	19	17.38	18.30	0.92	0.295	62	22.64	19.39	-3.25	0.372
Transferrin	4	0.57	0.71	0.14	0.301	21	1.01	1.08	0.07	0.503
Total Iron Binding Capacity	4	15.38	18.18	2.80	0.302	19	29.25	25.76	-3.49	0.453
Urine Leukocytes	11	0.23	0.46	0.23	0.302	33	0.47	0.59	0.12	0.552
Beta 2 Microglobulin	2	6.08	5.04	-1.04	0.310	13	7.52	7.60	0.09	0.906
Blood Urea Nitrogen	69	9.64	9.09	-0.56	0.324	199	10.32	10.82	0.50	0.305
Lipase	5	29.62	32.01	2.39	0.336	18	37.34	67.71	30.37	0.126
PLT	64	311.39	320.06	8.66	0.343	187	303.67	296.40	-7.27	0.321

Characteristic	Rivaroxaban_n	Rivaroxaban_Baseline Mean	Rivaroxaban_Follow-up Mean	Rivaroxaban_Mean Difference	Rivaroxaban_P value	Indobufen_n	Indobufen_Baseline Mean	Indobufen_Follow-up Mean	Indobufen_Mean Difference	Indobufen_P value
Urine pH	29	6.22	6.35	0.13	0.362	109	6.49	6.42	-0.07	0.406
Urine Glucose	23	0.74	0.54	-0.20	0.362	46	0.61	0.82	0.21	0.182
Creatine Kinase	14	248.36	158.46	-89.89	0.379	42	416.95	213.42	-203.54	0.191
Urine Urobilinogen	4	0.38	0.13	-0.25	0.391	17	0.15	0.50	0.35	0.029
Complement C4	12	0.28	0.26	-0.02	0.417	31	0.24	0.23	-0.01	0.518
Urine Specific Gravity	42	1.02	1.02	0.00	0.423	107	1.02	1.02	0.00	0.291
Anti_Streptolysin O	6	42.50	44.58	2.08	0.520	23	33.22	31.70	-1.52	0.718
Immunoglobulin A	15	1.99	1.93	-0.07	0.527	43	2.10	1.89	-0.21	0.004

Characteristic	Rivaroxaban_n	Rivaroxaban_Baseline Mean	Rivaroxaban_Follow-up Mean	Rivaroxaban_Mean Difference	Rivaroxaban_P value	Indobufen_n	Indobufen_Baseline Mean	Indobufen_Follow-up Mean	Indobufen_Mean Difference	Indobufen_P value
APTT	20	35.16	36.45	1.29	0.548	62	35.92	32.23	-3.69	0.011
Amylase	5	51.20	53.50	2.30	0.560	17	67.41	79.79	12.38	0.288
C Reactive Protein	10	25.08	45.25	20.17	0.568	34	16.02	37.28	21.26	0.099
GLU	62	5.83	5.57	-0.26	0.576	184	5.21	5.62	0.41	0.041
Transferrin Saturation	3	32.64	35.82	3.18	0.582	19	45.67	32.69	-12.97	0.101
Urine Microalbumin	2	1360.50	549.00	-811.50	0.599	18	621.09	551.45	-69.64	0.695
Urine Red Blood Cells_Microscopic	50	41.99	72.84	30.85	0.600	143	35.11	131.82	96.72	0.291

Characteristic	Rivaroxaban_n	Rivaroxaban_Baseline Mean	Rivaroxaban_Follow-up Mean	Rivaroxaban_Mean Difference	Rivaroxaban_P value	Indobufen_n	Indobufen_Baseline Mean	Indobufen_Follow-up Mean	Indobufen_Mean Difference	Indobufen_P value
Gamma-Glutamyl Transferase	67	55.02	52.14	-2.87	0.652	180	45.98	59.24	13.27	< 0.001
Potassium	72	3.92	3.89	-0.03	0.696	208	4.05	4.03	-0.03	0.540
FOBT	5	0.60	0.40	-0.20	0.704	15	0.60	0.43	-0.17	0.475
LDH	15	280.93	293.10	12.17	0.722	45	322.20	302.32	-19.88	0.423
Triglycerides	50	2.77	2.64	-0.13	0.730	139	2.63	2.31	-0.31	0.051
Plateletcrit	58	0.29	0.28	0.00	0.745	177	0.28	0.26	-0.02	0.002
Complement C3	13	1.11	1.08	-0.02	0.748	39	1.07	1.05	-0.01	0.742
Alkaline Phosphatase	68	78.09	77.05	-1.04	0.757	177	77.67	80.82	3.15	0.058

Characteristic	Rivaroxaban_n	Rivaroxaban_Baseline Mean	Rivaroxaban_Follow-up Mean	Rivaroxaban_Mean Difference	Rivaroxaban_P value	Indobufen_n	Indobufen_Baseline Mean	Indobufen_Follow-up Mean	Indobufen_Mean Difference	Indobufen_P value
Urine Ketones	11	0.27	0.23	-0.05	0.762	32	0.23	0.24	0.01	0.932
Alanine Aminotransferase	68	28.90	29.72	0.82	0.822	173	28.56	35.39	6.83	0.006
Sodium	68	141.51	141.60	0.09	0.832	201	140.77	141.27	0.50	0.106
CK-MB	14	25.29	24.78	-0.52	0.852	41	48.85	26.27	-22.58	0.193
Unsaturated Iron Binding Capacity	3	8.43	7.90	-0.53	0.909	16	18.86	17.38	-1.47	0.810
Prothrombin Activity	20	92.87	93.40	0.53	0.912	59	101.84	102.76	0.92	0.699
Urine Epithelial Cells_Microscopic	37	11.03	10.81	-0.22	0.957	110	7.31	9.50	2.19	0.279

Characteristic	Rivaroxaban_n	Rivaroxaban_Baseline Mean	Rivaroxaban_Follow-up Mean	Rivaroxaban_Mean Difference	Rivaroxaban_P value	Indobufen_n	Indobufen_Baseline Mean	Indobufen_Follow-up Mean	Indobufen_Mean Difference	Indobufen_P value
Absolute Eosinophil Count	60	0.14	0.14	0.00	0.963	176	0.21	0.15	-0.06	0.066
MCH	59	27.14	27.15	0.00	0.980	176	27.86	28.07	0.21	0.017

P-values for within-group comparisons (baseline vs. follow-up) were calculated using the paired t-test or the Wilcoxon signed-rank test, as appropriate

Supplementary Table S3. Complete Outputs of the Linear Mixed-effects Models for All Analyzed Laboratory Parameters, Including Covariate Adjustments.

Characteristic	N (Events)	Main Effect Estimate	Main Effect SE	Main Effect t	Main Effect P	Interaction Estimate	Interaction SE	Interaction t	Interaction P
Prealbumin	469 (167)	-21.56	12.36	-1.74	0.082	56.00	12.92	4.34	< 0.001
Globulin	474 (170)	-3.00	0.64	-4.70	< 0.001	2.32	0.52	4.46	< 0.001
Urine Occult Blood	409 (142)	0.08	0.11	0.75	0.451	-0.37	0.10	-3.94	< 0.001
T4	212 (49)	-14.08	4.64	-3.03	0.003	16.51	4.00	4.13	< 0.001
Leucine Aminopeptidase	469 (167)	1.16	2.20	0.53	0.599	4.80	1.30	3.69	< 0.001
FT4	212 (49)	-1.63	0.84	-1.94	0.054	3.13	0.84	3.73	< 0.001
MPV	464 (174)	-0.10	0.11	-0.96	0.335	-0.32	0.09	-3.67	< 0.001
Urine Dipstick Protein	374 (137)	0.52	0.11	4.85	< 0.001	-0.39	0.11	-3.61	< 0.001
Low-Density Lipoprotein	388 (135)	0.82	0.32	2.60	0.010	-0.91	0.26	-3.53	0.001

Characteristic	N (Events)	Main Effect Estimate	Main Effect SE	Main Effect t	Main Effect P	Interaction Estimate	Interaction SE	Interaction t	Interaction P
Cholinesterase	474 (170)	1229.94	315.42	3.90	< 0.001	-656.47	186.35	-3.52	0.001
Total Protein	476 (172)	-6.11	1.29	-4.73	< 0.001	4.06	1.17	3.47	0.001
Total Cholesterol	396 (140)	1.29	0.39	3.29	0.001	-0.99	0.29	-3.42	0.001
Bicarbonate	518 (193)	1.35	0.46	2.95	0.003	-1.49	0.46	-3.22	0.001
C Reactive Protein	223 (48)	-6.93	6.83	-1.02	0.311	19.37	6.13	3.16	0.002
ALB	479 (171)	-3.70	0.84	-4.42	< 0.001	2.26	0.76	2.98	0.003
PDW	464 (174)	-0.24	0.31	-0.77	0.441	0.95	0.34	2.76	0.006
Plateletcrit	464 (174)	0.01	0.01	0.83	0.410	-0.02	0.01	-2.75	0.006
T3	212 (49)	-0.14	0.09	-1.59	0.113	0.17	0.07	2.59	0.010
Apolipoprotein B	333 (106)	0.19	0.08	2.31	0.021	-0.17	0.07	-2.54	0.011

Characteristic	N (Events)	Main Effect Estimate	Main Effect SE	Main Effect t	Main Effect P	Interaction Estimate	Interaction SE	Interaction t	Interaction P
Urine Protein Concentration	383 (96)	2.13	0.94	2.28	0.023	-1.87	0.75	-2.49	0.013
Calcium	525 (193)	-0.06	0.02	-2.93	0.004	0.05	0.02	2.31	0.021
Lipoprotein a	325 (102)	185.70	92.86	2.00	0.046	-168.01	72.95	-2.30	0.022
High-Density Lipoprotein	388 (135)	-0.01	0.09	-0.16	0.876	0.17	0.08	2.14	0.033
Uric Acid	516 (193)	-29.74	14.07	-2.11	0.035	27.45	13.51	2.03	0.043
Urine Small Round Epithelial Cells_Microscopic	168 (48)	0.23	0.10	2.32	0.021	-0.22	0.12	-1.95	0.052
PLT	475 (175)	13.65	11.78	1.16	0.247	-17.40	9.11	-1.91	0.057
FT3	212 (49)	-0.15	0.27	-0.58	0.562	0.45	0.24	1.90	0.059
Serum Triglycerides	396 (140)	0.50	0.19	2.62	0.009	-0.33	0.18	-1.81	0.071
Absolute Lymphocyte Count	475 (175)	0.17	0.12	1.34	0.180	0.20	0.13	1.56	0.119

Characteristic	N (Events)	Main Effect Estimate	Main Effect SE	Main Effect t	Main Effect P	Interaction Estimate	Interaction SE	Interaction t	Interaction P
Transferrin Saturation	191 (37)	9.13	4.41	2.07	0.039	-4.60	2.99	-1.54	0.125
D-Dimer	194 (63)	-0.91	0.85	-1.07	0.286	1.02	0.72	1.43	0.156
Serum Inorganic Phosphorus	524 (192)	-0.02	0.04	-0.60	0.552	0.05	0.04	1.41	0.159
IBIL	474 (170)	-0.36	0.31	-1.18	0.237	0.38	0.27	1.40	0.163
TBIL	474 (170)	-0.47	0.43	-1.07	0.284	0.53	0.38	1.39	0.165
Absolute Eosinophil Count	475 (175)	0.08	0.03	2.64	0.009	-0.05	0.03	-1.38	0.170
Ferritin	145 (27)	111.59	94.07	1.19	0.237	56.22	42.26	1.33	0.186
Eosinophil Percentage	475 (175)	0.61	0.30	2.01	0.044	-0.36	0.28	-1.28	0.201
DBIL	474 (170)	-0.10	0.18	-0.58	0.564	0.20	0.15	1.27	0.207
FIB	237 (71)	0.79	0.31	2.57	0.011	-0.30	0.26	-1.16	0.249

Characteristic	N (Events)	Main Effect Estimate	Main Effect SE	Main Effect t	Main Effect P	Interaction Estimate	Interaction SE	Interaction t	Interaction P
GLU	471 (171)	-0.30	0.31	-0.96	0.337	0.36	0.32	1.14	0.253
Procalcitonin	81 (27)	-6.76	4.98	-1.36	0.177	5.40	4.74	1.14	0.257
Cystatin C	428 (166)	-0.07	0.16	-0.40	0.693	0.09	0.08	1.13	0.259
TSH	214 (49)	-0.13	1.31	-0.10	0.918	-0.88	0.83	-1.07	0.287
APTT	237 (71)	2.04	1.71	1.19	0.235	-1.83	1.72	-1.06	0.290
Serum Gamma-Glutamyl Transferase	474 (170)	2.10	10.30	0.20	0.839	4.94	4.70	1.05	0.294
Urine Hyaline Casts_Microscopic	168 (48)	0.68	0.61	1.11	0.268	-0.55	0.54	-1.03	0.307
Immunoglobulin A	266 (58)	-0.20	0.16	-1.27	0.204	-0.06	0.06	-1.01	0.313
Urine Other Casts_Microscopic	443 (148)	1.30	0.72	1.81	0.070	-0.85	0.85	-1.00	0.319
Antithrombin III	129 (49)	-3.50	5.04	-0.70	0.488	4.40	4.56	0.96	0.337

Characteristic	N (Events)	Main Effect Estimate	Main Effect SE	Main Effect t	Main Effect P	Interaction Estimate	Interaction SE	Interaction t	Interaction P
Rheumatoid Factor	154 (29)	3.69	8.18	0.45	0.653	-5.98	6.28	-0.95	0.343
ApoA1	286 (97)	0.08	0.07	1.27	0.204	0.06	0.06	0.95	0.345
Urine Other Crystals_Microscopic	168 (48)	4.44	3.50	1.27	0.205	-3.70	4.45	-0.83	0.408
Serum Sodium	528 (196)	0.42	0.39	1.09	0.277	-0.30	0.40	-0.75	0.452
White Blood Cell Count	475 (175)	0.93	0.53	1.75	0.081	0.35	0.52	0.68	0.498
Serum Aspartate Aminotransferase	476 (170)	3.13	17.05	0.18	0.854	-12.99	21.56	-0.60	0.547
Absolute Basophil Count	475 (175)	0.00	0.00	0.96	0.340	0.00	0.00	-0.53	0.594
MCV	475 (175)	1.39	0.93	1.50	0.135	-0.16	0.31	-0.52	0.607
Adenosine Deaminase	476 (172)	0.79	0.75	1.05	0.293	-0.29	0.67	-0.43	0.667
Urine Granular Casts_Microscopic	168 (48)	-0.19	0.51	-0.36	0.716	0.23	0.57	0.39	0.695

Characteristic	N (Events)	Main Effect Estimate	Main Effect SE	Main Effect t	Main Effect P	Interaction Estimate	Interaction SE	Interaction t	Interaction P
Absolute Monocyte Count	475 (175)	0.02	0.04	0.53	0.597	0.02	0.04	0.35	0.729
Glomerular Filtration Rate	428 (166)	-1.02	2.83	-0.36	0.718	-0.56	1.80	-0.31	0.756
Absolute Neutrophil Count	475 (175)	0.64	0.49	1.32	0.187	0.14	0.48	0.29	0.769
Albumin/Globulin Ratio	474 (170)	-0.03	0.04	-0.77	0.441	-0.01	0.03	-0.29	0.773
Neutrophil Percentage	475 (175)	0.22	1.34	0.17	0.868	0.33	1.45	0.23	0.822
RDW-CV	475 (175)	-0.42	0.30	-1.39	0.164	0.04	0.18	0.21	0.832
Monocyte Percentage	475 (175)	-0.50	0.31	-1.63	0.104	-0.06	0.33	-0.18	0.857
Urine Glucose	454 (148)	0.24	0.06	3.74	< 0.001	-0.01	0.06	-0.15	0.884
MCH	475 (175)	0.67	0.36	1.87	0.062	0.02	0.11	0.14	0.888
Serum Alanine Aminotransferase	476 (170)	1.87	11.43	0.16	0.870	-1.90	13.95	-0.14	0.892

Characteristic	N (Events)	Main Effect Estimate	Main Effect SE	Main Effect t	Main Effect P	Interaction Estimate	Interaction SE	Interaction t	Interaction P
Serum Chloride	528 (196)	0.30	0.56	0.55	0.584	-0.07	0.53	-0.13	0.897
Hematocrit	475 (175)	2.31	0.83	2.79	0.006	-0.05	0.51	-0.10	0.919
HGB	475 (175)	8.27	2.85	2.91	0.004	0.13	1.67	0.08	0.939
Red Blood Cell Count	475 (175)	0.20	0.10	1.98	0.048	0.00	0.06	0.06	0.954
Basophil Percentage	475 (175)	0.00	0.03	0.10	0.921	0.00	0.03	0.03	0.976

The following tables present the full fixed-effects output from the linear mixed-effects models for each laboratory parameter. The model included treatment group (Indobufen vs. Control), time (Follow-up vs. Baseline), their interaction, and adjustments for age, sex, and major concomitant medications. Patient ID was included as a random effect.
